# Optimal spatial evaluation of a pro rata vaccine distribution rule for COVID-19

**DOI:** 10.1038/s41598-023-28697-8

**Published:** 2023-02-07

**Authors:** François M. Castonguay, Julie C. Blackwood, Emily Howerton, Katriona Shea, Charles Sims, James N. Sanchirico

**Affiliations:** 1 Department of Agricultural and Resource Economics, University of California, Davis, Davis, CA 95616 USA; 2 Department of Mathematics and Statistics, Williams College, Williamstown, MA 01267 USA; 3 Department of Biology and Center for Infectious Disease Dynamics, Pennsylvania State University, University Park, PA 16802 USA; 4 Howard H. Baker Jr. Center for Public Policy and Department of Economics, University of Tennessee, Knoxville, Knoxville, TN 37996 USA; 5 Department of Environmental Science and Policy, University of California, Davis, Davis, CA 95616 USA; 6 Resources for the Future, Washington, DC 20036 USA

**Keywords:** Epidemiology, Preventive medicine, Health policy

## Abstract

The COVID-19 Vaccines Global Access (COVAX) is a World Health Organization (WHO) initiative that aims for an equitable access of COVID-19 vaccines. Despite potential heterogeneous infection levels across a country, countries receiving allotments of vaccines may follow WHO’s allocation guidelines and distribute vaccines based on a jurisdictions’ relative population size. Utilizing economic—epidemiological modeling, we benchmark the performance of this pro rata allocation rule by comparing it to an optimal one that minimizes the economic damages and expenditures over time, including a penalty representing the social costs of deviating from the pro rata strategy. The pro rata rule performs better when the duration of naturally- and vaccine-acquired immunity is short, when there is population mixing, when the supply of vaccine is high, and when there is minimal heterogeneity in demographics. Despite behavioral and epidemiological uncertainty diminishing the performance of the optimal allocation, it generally outperforms the pro rata vaccine distribution rule.

## Introduction

Now that several vaccines against coronavirus disease 2019 (COVID-19) have obtained the World Health Organization’s (WHO) emergency use listing, policymakers around the globe are deciding how to allocate the limited supplies within their boundaries. While the vaccination campaign of developed countries like the U.S. and the U.K. has stagnated, developing countries in Asia and Africa still face major vaccine supply issues. In anticipation of this problem, WHO and other partners created “COVID-19 Vaccines Global Access” (COVAX)—an initiative that aims for an equitable access of COVID-19 vaccines. The scientific literature has addressed the question of vaccine allocation, but most of it has focused on demographic considerations within one jurisdiction^1–3^ or on a global scale^4–6^. This prior work has made important contributions to the debate. A missing piece in the allocation question, however, is how to divide up limited quantities across jurisdictions (e.g., state/provinces, counties/regions) that might have different demographic and epidemiological characteristics. In the U.S., the National Academies of Sciences, Engineering, and Medicine (NASEM) recommends that vaccines are allocated to jurisdictions based on their relative population size “in the interest of speed and workability”^7^ and WHO applies a similar pro rata distribution rule with its COVAX program^8^. Countries whose vaccination campaign depends on COVAX provision could also apply a similar pro rata distribution rule within their boundaries.

In this paper, we explore the economic and epidemiological trade-offs of such a simple allocation rule, for various levels of vaccine scarcity. We derive the optimal allocation as a benchmark to compare with the pro rata rule and we investigate how robust the optimal allocation is to incorrect behavioral (i.e., compliance to nonpharmaceutical interventions) and epidemiological (i.e., the duration of immunity) assumptions. The optimal allocation mimics a case where jurisdictions are allowed to trade vaccines amongst themselves, or COVAX distributes the vaccine, so that the jurisdiction that would yield the greatest benefits of the vaccine will obtain it. While in theory the pro rata rule cannot perform better than the optimally derived rule, this is not necessarily true in practice because key factors may be unknown to policymakers. We also investigate how various vaccine effectiveness—mimicking the wide range of effectiveness of the vaccines available for distribution (for the lower and upper end of the spectrum, see^9,10^ respectively) and the reduced effectiveness of vaccines when faced with emerging variants of concern^11,12^—affect the optimal allocation. The benchmark optimal rule we consider minimizes the economic costs from health-related damages, vaccine expenditures, and a workability cost imposed on the planner for deviating from the pro rata rule.

In a world where two jurisdictions are identical in terms of population, the pro rata rule would divide the limited supply equally between the jurisdictions. However, it is much more likely that two jurisdictions, even if equally sized, have heterogeneous levels of infections (e.g., in terms of cases) at the time a country’s central government receives an allotment of vaccines. Based on prior literature on spatial-dynamics of disease management, heterogeneity in infection levels may lead to significant deviations between the optimal spatial allocation and the pro rata distribution potentially leading to greater economic costs and worse public health outcomes (see^13^ for example).

Mechanisms leading to heterogeneous infection include the timing of the outbreak, demographic characteristics of the population (e.g., age structure^14^ and essential worker status^15^), and the implementation of and compliance with preventative nonpharmaceutical interventions; see^16^ for more details on how SARS-CoV-2 (i.e., the virus that causes COVID-19) prevalence may vary across space. While compliance to preventive measures may seem independent from vaccine allocation, it affects the initial conditions (i.e., the conditions before the vaccine is allotted to the country) and the conditions under which the limited supplies will be allocated within the country. For example, compliance to shelter-in-place and travel restrictions results in little to no movement of the virus from one jurisdiction to another. When regions are non-interacting, Brandeau et al.^17^ show for a general susceptible-infected-susceptible (SIS) model that the optimal allocation of resources depends on numerous intrinsic factors, including the size of the populations of each jurisdiction and the level of infection at the time of the vaccine allotment. When regions are interacting, Rowthorn et al.^18^ show when there is no immunity (i.e., in an SIS model) that treatment should be preferentially directed towards the region that has the lower level of infection. However, this result only holds in general over the entire time horizon and priority may switch from one jurisdiction to another over the course of an outbreak. Importantly, the time at which priority switches from one jurisdiction to another is critical, and other researchers^19^ have found that missing the switch point may lead to suboptimal outcomes. While these results indicate that a fixed pro rata distribution rule is less cost-effective in an SIS model, whether compliance to travel restrictions makes the pro rata rule relatively more cost-effective in the case of COVID-19 is an open question.

Our findings illustrate that the vaccines should be optimally allocated over time depending on: (i) if the jurisdiction has a lower or higher infection level at the time of the vaccine allotment, (ii) if immunity is permanent (see Zhou et al.^20^) or temporary (Gersovitz and Hammer^21^ already pointed out that the optimal allocation is conditional on the duration of immunity), (iii) whether there is compliance to travel restrictions or not, (iv) the amount of vaccine available, and (v) the average demographic characteristics of the population (mimicking age structure and essential worker status). We make a simplifying assumption and proxy variability in demographics by assuming that the population of one jurisdiction has a higher case-fatality ratio (e.g., an older population^14^) or a higher contact rate (e.g., a population with more essential workers^15^) than the other. We find that a pro rata distribution rule—which prioritizes equity of distribution—performs relatively better when immunity is temporary, when there is noncompliance to travel restrictions, when the vaccine supply is high, and when there is minimal heterogeneity in demographic characteristics. On the downside, allocating a vaccine based on a pro rata distribution rule generally leads to an over-utilization in jurisdictions where disease prevalence is higher, an under-utilization in jurisdictions where disease prevalence is lower, and overall a higher number of cumulative cases. Whether these inefficiencies outweigh the “speed and workability”^7^ inherent in simple allocation rules is an important question for policymakers. Our research can aid in that discussion by illuminating the trade-offs involved in such complex epidemiological, economic, and social decisions by providing optimal benchmarks from which to compare allocation rules.

While the optimal allocation is conditional on a number of factors mentioned above, the science remains unresolved on the duration of immunity to SARS-CoV-2, and it is difficult to anticipate and subsequently estimate the extent to which populations in different jurisdictions comply with the travel restrictions. On the other hand, the pro rata rule has the advantage of being based on easily observable factors (i.e., a jurisdiction’s population size). To gain insights into the robustness of optimal and pro rata policies in the presence of such uncertainties, we investigate the economic and public health consequences that could occur if we design an optimal policy or evaluate the performance of the pro rata rule under a set of assumptions on immunity and compliance that turn out to be incorrect.

We make a number of contributions to the literature. First, we develop a method to assess the performance of a pro rata vaccine distribution rule based on relative population size: we develop an economic—epidemiological model and solve for the optimal allocation of vaccines over time to minimize the economic costs from health-related damages, vaccine expenditures, and a workability cost imposed on the planner for deviating from the pro rata rule. We then compare the outcome of such optimal rule with the outcome of the pro rata rule. Prior literature considering the trade-offs involved with rules of thumb (such as the pro rata rule we consider in this paper) does not consider that deviating from them entails potential workability costs (see, for example,^22^). Second, we consider how the performance of a pro rata rule is influenced by compliance with preventative nonpharmaceutical interventions (i.e., travel restrictions) and various demographics (we proxy for age structure and essential worker status). Third, and perhaps most importantly, we show that in general, optimal rules are robust to incorrect epidemiological assumptions (about the duration of immunity) but that incorrect behavioral assumptions (about compliance to travel restrictions) and the presence of demographic heterogeneities (in the age structure of jurisdictions) can lead to a considerably poorer performance of the optimal allocation (i.e., higher cumulative cases) despite still generally outperforming the pro rata rule.

The paper is divided as follow. In “Materials and methods”, we detail the different types of interventions, we present the components of the economic—epidemiological model, and detail the technique used to analyse the allocation question. “Results” presents the results while “Discussion” concludes the paper.

## Materials and methods

We develop an economic—epidemiological model to describe the dynamics of SARS-CoV-2. The model captures a situation where a central planning agency (e.g., the central government) must decide when and how much of the scarce vaccines to allocate to two jurisdictions where disease burden (i.e., infection level) is heterogeneous at the moment it receives an allotment of vaccine. We assume that the objective of the central planner is to minimize costs across both jurisdictions, including damages associated with the morbidity and deaths of infected individuals, the expenditures related to the pharmaceutical intervention, and a penalty cost mimicking the increased workability costs incurred for any deviation from the pro rata distribution rule. The dynamics of SARS-CoV-2 are modeled using an SEIR epidemiological model, which tracks the change over time of the susceptible (S), exposed (E), infected (I), and recovered (R) populations for two separate jurisdictions (see Appendix A for more details on the calibration of the model). We note that while we generally talk about these jurisdictions as being two different states, they can very well represent any two sub-national jurisdictions like provinces or territories, and even counties or regions within one sub-national jurisdiction.

### Modelling different types of intervention

There are two different types of interventions we consider: travel restrictions and vaccines. We assume that travel restrictions affect both jurisdictions simultaneously (e.g., by an order from the central government), and that the populations either comply perfectly or imperfectly to the travel restrictions (for examples of optimal lockdown policies see, e.g.,^23,24^). When compliance is perfect, individuals in different jurisdictions do not interact with each other and thus susceptible individuals can only get infected by being in contact with some infected individual in their own jurisdiction. When compliance is imperfect, susceptible individuals from one jurisdiction can also travel to the other jurisdiction where they can be in contact with infected individuals, or infected individuals from one jurisdiction can travel to the other jurisdiction and infect susceptible individuals there; this discrete shift in the number of contacts effectively increases the transmissibility of the virus (see Appendix A more details).

We assume that the analysis starts when the central planning agency has received an allotment of vaccines and will keep receiving a continuous allotment of vaccines. For simplicity, the amount of available vaccine is assumed to be exogenous to the model and fixed over time, which is likely given the short time frames we consider in the paper (4 months). However, we consider different levels of vaccine allotments, or capacity, to investigate how different levels of vaccine scarcity may affect their optimal allocation. In our model, vaccines reduce the pool of susceptible individuals by providing them with immunity from the virus, as early evidence suggests that vaccines could be transmission blocking in addition to preventing severe disease^25^.

### Model of disease transmission

**Figure 1:**
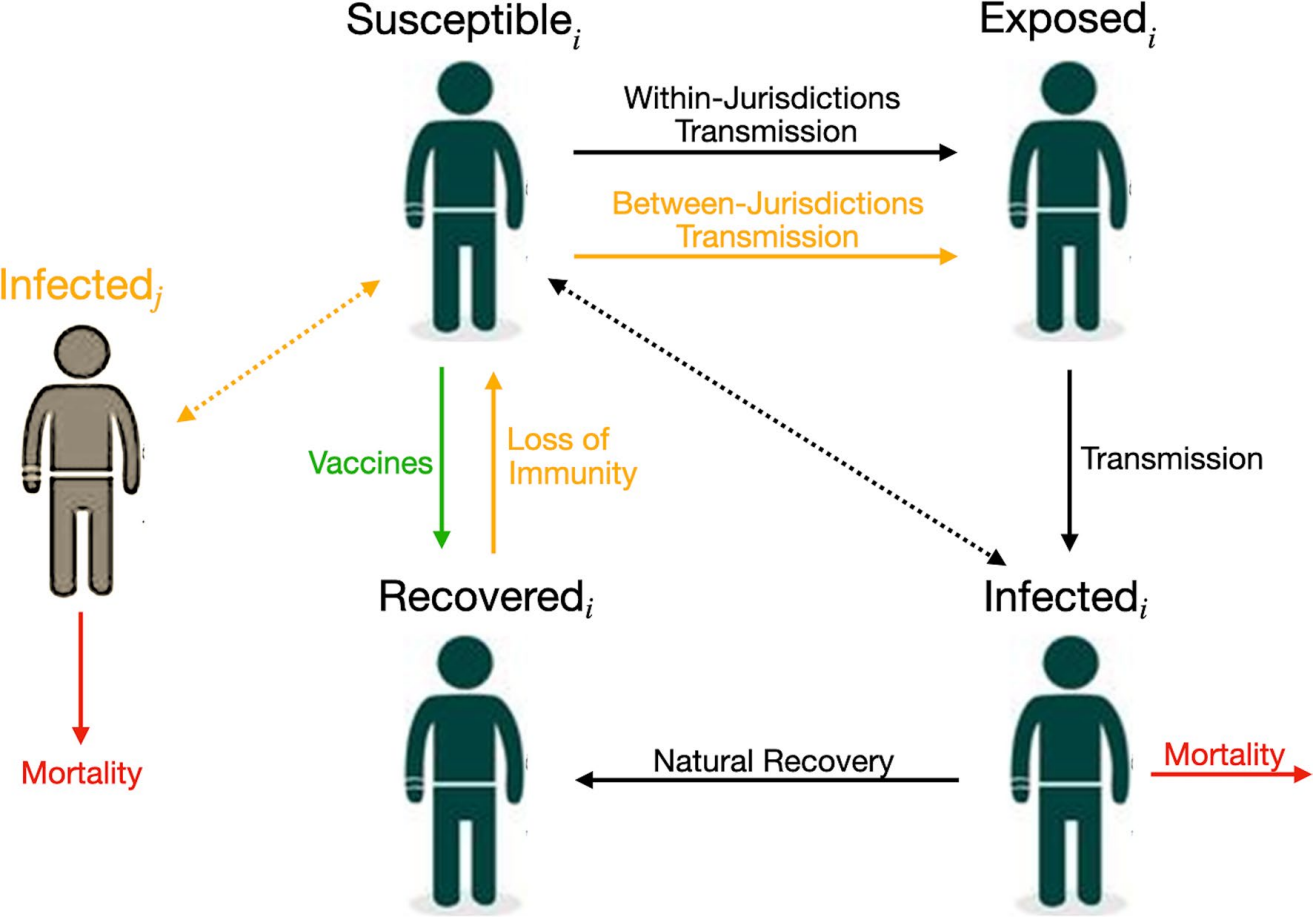
Model interventions and disease transmission pathways for our model of COVID-19. The full lines represent the transition between, or out of, compartments while the dotted lines represent contact between susceptible and infected individuals. Black lines represent situations that do not vary, while yellow lines represent key factors that we vary in our model to see how they impact our results. The green line represents the vaccines and the red line represents mortality.

We use a frequency-dependent^26^ susceptible—exposed—infected—recovered (SEIR) model that describes the dynamics of COVID-19 in two separate jurisdictions $i=1,2$ (e.g., states/provinces or counties/regions); each jurisdiction contains a population of $N_i$ individuals that is either susceptible, exposed, infected, or recovered (see Fig. 1). We also consider scenarios where immunity is temporary (i.e., lasts 6 months, for more details see^27^), thus also using an SEIR-Susceptible (SEIRS) model (for COVID-19 applications see, e.g.,^28–31^). In such scenarios, the $R_i$ recovered individuals are immune for a mean period of $\frac{1}{\omega }$ months.

In each jurisdiction *i*, the $S_i$ susceptible individuals are in contact with the $I_i$ infected individuals of their own jurisdiction at a rate of $\beta _{ii}$ and are in contact with the $I_j$ infected individuals of the other jurisdiction at a rate of $\beta _{ij}$. We assume $\beta _{ij}=0$ (i.e., no mixing between jurisdictions) when there is perfect compliance to travel restrictions, and $\beta _{ij}>0$ if not. To highlight the role of travel restriction compliance and initial disease burden, we initially assume that the contact rate is identical across jurisdictions, meaning that $\beta _{11}=\beta _{22}=\beta _{ii}$ and $\beta _{12}=\beta _{21}=\beta _{ij}$ (further, we relax this assumption and investigate the optimal allocation when there is heterogeneity in the contact rate). We assume there is no permanent migration of individuals from one jurisdiction to another (see for instance^32^) in the sense that individuals who do not comply with travel restrictions do not permanently move to the other state, but instead travel to it temporarily. An implication is that we are assuming that the two jurisdictions are close enough for such travel and mixing to be economically feasible.

We model the control variables for vaccines as non-proportional controls, i.e., available in a constant amount each month^2,18,33^. The change in susceptible individuals is

1 $$\begin{aligned} \dot{S_i} = \omega R_i - \beta _{ii} S_i \frac{I_i}{N_i} - \beta _{ij} S_i \frac{I_j}{N_j} - q_V u_{V_i}, \end{aligned}$$

where $u_{V_i}$ represents the number of individuals being treated via vaccine in a given time period (i.e., a month) in Jurisdiction *i*, and $q_V$ represents the effectiveness of the vaccine (note that we look at the low end of vaccine effectiveness to be conservative; see “Sensitivity analyses” for more details). We note that our model does not distinguish between individuals whose vaccine has failed and those who have not been vaccinated at all. As such, individuals with vaccine failure can be re-vaccinated in subsequent months.

After being infected, susceptible individuals transition into the exposed class $E_i$ where the disease remains latent for a mean period of time of $\frac{1}{\sigma }$, before the onset of infectiousness. The change in the number of exposed individuals is

2 $$\begin{aligned} \dot{E_i} = \beta _{ii} S_i \frac{I_i}{N_i} + \beta _{ij} S_i \frac{I_j}{N_j} - \sigma E_i. \end{aligned}$$

Exposed individuals eventually become infectious for a mean period of time of $\frac{1}{\gamma +\varphi _i}$ and in turn can infect susceptible individuals. Infected individuals either recover naturally from the disease at a rate of $\gamma$ or die from complications related to infection at a disease induced mortality rate of $\varphi _i$. In our base case we assume identical disease induced mortality rates across jurisdictions, i.e., $\varphi _1 = \varphi _2=\varphi$ but we also investigate the optimal allocation when $\varphi _1 \ne \varphi _2$. The growth of the infected individuals is

3 $$\begin{aligned} \dot{I_i} = \sigma E_i - \gamma I_i - \varphi _i I_i. \end{aligned}$$

The recovered population $R_i$ includes individuals that recover naturally from the disease at a rate of $\gamma$ and the individuals that are successfully vaccinated every month ($q_V u_{V_i}$); if immunity is temporary ($\omega >0$), a fraction of the recovered will leave this compartment. Our model does not distinguish between vaccine-acquired immunity and naturally-acquired immunity. The number of recovered individuals in Jurisdiction *i* thus changes according to

4 $$\begin{aligned} \dot{R_i} = \gamma I_i + q_V u_{V_i} - \omega R_i. \end{aligned}$$

At any instant in time, we have that $N_i = S_i + E_i + I_i + R_i$, which in turn implies that the growth of the population over time is

5 $$\begin{aligned} \dot{N_i} = - \varphi _i I_i. \end{aligned}$$

In keeping with much of the previous economic epidemiology literature^21^, we have omitted natural births and non-COVID-related deaths due to the short time frame of our model (4 months) and assume reductions in international travel^34^ effectively lead to a closed population (i.e., there is no exogenous importation of infected individuals). See Appendix A for more details about the parameterization of the epidemiological model.

### Modeling the pro rata distribution rule

We model a pro rata rule that favors “speed and workability”^7^. We follow the NASEM^7^ and WHO^8^ principle and impose the pro rata vaccine distribution based on relative population size. Specifically, the rule for Jurisdiction *i* is that

6 $$\begin{aligned}&u_{V_i} \le \bigg ( \frac{N_i}{N_1 + N_2}\bigg ) \bar{u}_{V}, \end{aligned}$$

where $\bar{u}_{V}$ is the limited amount of vaccine allotted to the central government. When the population sizes are the same, the pro rata distribution rule will divide equally the limited doses to the two jurisdictions.

In the scenarios where we consider the pro rata distribution rule, we model the allocation rule as an inequality because towards the end of the horizon after periods of vaccinations, the level of susceptible in the population may be such that the limited supply of vaccines is not an issue. Other rules of thumb are possible, such as, allocate all to the largest or smallest population^22^, but we concentrate on the one currently being advocated for by NASEM^7^ and WHO^8^.

### Model of economic costs

The model of economic costs include damages related to morbidity and deaths, costs spent on the vaccines, and the workability cost described above that is incurred for any deviation from the pro rata rule. Damages represent consequences related to a temporary disability associated with severe or critical symptoms, and loss of life in the worst cases. While we have chosen to use infections as the primary indicator of damages, alternatives such as disease severity could also be considered. The damages are assumed to be linear and additively separable across jurisdictions, meaning that they are identical across individuals and across jurisdictions. The marginal value of damages (i.e., the damages associated with the death of one individual) is assumed to be constant over time and given by the value of a statistical life (VSL) that the U.S. Environmental Protection Agency^35^ uses (see Appendix A for more details on the parameterization). Damages incurred from a temporary disability associated with severe or critical symptoms can be compared to deaths via some disability weight *w*; given we found no published disability values associated with COVID-19, we follow the literature (see for instance^36^), and use the disability value associated with lower respiratory tract infections. The damage function for Jurisdiction *i* is

7 $$\begin{aligned} c_i(I_i) = (w+\varphi _i) c I_i, \end{aligned}$$

where *c* is the damage parameter associated infectious individuals. While we motivate this cost parameter with disease-related disability and loss of life, other potential pathways for costs varying with infection levels include direct health costs^37^, psychological distress^38^, or the cost of shutting down the economy in response to increased infections^39^.

We model a scenario where the central planner is focused on the allocation of vaccines where the costs for its development have already been incurred. This implies that vaccine development costs have already been utilized (in technical terms we say that the costs are sunk) and therefore do not affect the decision of the central planning agency. We model the vaccination cost as linear, where the cost parameter represents the cost of vaccinating one individual. The vaccine cost function is denoted $c_{V_i}(u_{V_i})$, with $i=1,2$. We assume that the vaccination cost is additively separable across jurisdictions such that we denote the cost of vaccinating $u_{V_i}$ individuals as

8 $$\begin{aligned} c_{V_i} (u_{V_i}) = c_{V} {u_{V_i}} \quad \text { for }\quad i=1,2, \end{aligned}$$

where $c_{V}$ represents the cost of treating one individual via vaccine. Note that calibration of the cost parameter is based on current vaccine prices (see Appendix A for more details about the parameterization of the economic model), but it could also represent the cost the central planner pays to administer the vaccine.

We assume that the central planning agency incurs a workability cost representing the social (transaction) costs of deviating from the pro rata rule (for another application of this concept, see^40^). The workability cost function is:

9 $$\begin{aligned}&c_{A}(u_{V_1},u_{V_2}, N_1, N_2) = c_{A} \Bigg ( \bigg ( \frac{N_2}{N_1 + N_2}\bigg ) u_{V_1} - \bigg (\frac{N_1}{N_1 + N_2}\bigg ) u_{V_2}\Bigg )^2, \end{aligned}$$

where $c_A$ is the parameter associated with the workability cost. When the gains from deviating from the pro rata distribution rule (i.e., a reduction in damages in one jurisdiction) outweigh the costs (i.e., an increase in damages in the other jurisdiction and the increased workability costs incurred), the central planning agency will prioritize this allocation as it will lead to lower total costs. By imposing the pro rata rule ex ante, the decision-maker is essentially assuming that this workability cost is infinite. Everything else being equal, we expect that the presence of the workability cost will push the optimal allocation towards the pro rata rule (see Supplementary Fig. S18 in the appendix for a sensitivity analysis of our results to the workability cost parameter). Therefore, when we do find deviations, we need to consider that the deviations and trade-offs would be greater if workability costs were smaller.

### Planner’s objective

In optimal control theory, the best, or optimal, path of the control variables (here the allocation of the limited supply of vaccines) is conditional on the objective of the central planning agency. We assume that the objective is to minimize the economic damages and the costs of the pharmaceutical intervention across jurisdictions over time, rather than a solely epidemiological objective (see for instance^18^). The objective function is the net present value of damages, expenditures related to vaccination, and the workability cost over an exogenously determined planning horizon (4 months). Specifically, the planner’s objective is:

10 $$\begin{aligned} \min _{u_{V_1}, u_{V_2}}&\int _{0}^T e^{-rt}\Big \{ c_1(I_1) + c_2(I_2) + c_{V_1}(u_{V_1}) + c_{V_2}(u_{V_2}) + c_{A}(u_{V_1},u_{V_2}, N_1, N_2) \Big \} \textrm{d}t, \end{aligned}$$

where *r* is the monthly discount rate. The planner solves equation (10) over a fixed time interval, *T*, subject to equations (1), (2), (3), (4), (5), along with constraints on availability of vaccines ($u_{V_1} + u_{V_2} \le \bar{u}_{V}$), non-negativity conditions, physical constraints on vaccines, initial disease burdens in each jurisdictions, and free endpoints (see discussion on terminal conditions in the next section). In the pro rata rule scenarios, we also impose Eq. (6).

### Initial and terminal conditions

The disease burden in each jurisdiction at the beginning of the time horizon (i.e., in $t=0$ when the allotment of vaccine is received for the first time) is calibrated using the epidemiological model (Eqs. (1), (2), (3), (4), and (5)). At the beginning of the outbreak, we assume that, in each jurisdiction, there is one exposed individual in an otherwise entirely susceptible population of 10 million individuals, and that populations of the different jurisdictions comply with the travel restrictions. The only difference between the two jurisdictions is that the outbreak started one week earlier in Jurisdiction 2. We simulate the outbreak for approximately nine months to yield the initial conditions; see Appendix B for more details. In a later section, we also proxy for heterogeneity in demographic characteristics (by varying case-fatality ratio and contact rate) and we modify the initial conditions accordingly assuming an identical timing in the outbreak of the disease.

We impose no conditions on the number of susceptible, exposed, infected, and recovered individuals at the end of the planning horizon; in technical terms, we say that the state variables are free (see Appendix B for more details). Under our free endpoint conditions, there is a transversality condition (i.e., a necessary condition for the vaccine allocation to be optimal) for each state variable that requires the product of the state variable ($S_i, E_i, I_i, R_i$ or $N_i$) and its corresponding costate variable (i.e., the shadow value, or cost, associated with the state variable) is equal to zero. Hence, at the end of the time horizon, either the state variable equals zero, the shadow value associated with the state variable equals zero, or both. In any case, allowing state variables to be free guarantees that the terminal levels of the state variables are optimally determined. Another possible assumption could be that over a fixed interval we find the optimal policy such that at the end of the horizon there is a given percent reduction in infected or susceptible individuals. Our approach nests this more restricted scenario.

## Results

To assess the performance of the pro rata rule relative to the optimal allocations of vaccine over time, we numerically solve the optimal control problem across three different scenarios: no controls, optimal vaccine allocation, and pro rata vaccine allocation. We investigate how to allocate vaccines by mapping out the different allocation rules for different immunity—travel restrictions—capacity scenarios. Any deviation from the pro rata rule is optimal despite incurring the workability cost. As the workability cost parameter $c_A$ goes to zero, the problem becomes linear in the controls where the optimal allocations in linear problems follow singular solutions. We use pseudospectral collocation to solve for the optimal dynamics of vaccine and infection over time, which converts the continuous time optimal control problem into a constrained non-linear programming problem solving for the coefficients of the approximating polynomials at the collocation nodes (see^41,42^ for other applications, and see Appendix B for more details on this technique).

We present the results for our preferred specification of the parameters (i.e., following what was estimated in the literature; see details in Appendix A) and for the case where immunity is permanent and the case where immunity is temporary. We detail the performance of the pro rata rule relative to the optimal allocation (henceforth, the optimal deviation) based on whether the populations of the different jurisdictions are compliant to travel restrictions or not, and for different levels of vaccine capacity constraints. The total available quantity of vaccine in a given time period (i.e., a month; $\bar{u}_V$) is based on a certain percentage (5%, 10%, or 15%) of the total population size. We focus our analysis on the period of time when the scarcity of the vaccine constraint is binding, as once the constraint relaxes the allocation question becomes moot. We further investigate how two key parameters affect these results and finally focus on cases where key parameters are unknown to the policymaker.

### Base case: when decisions are made with perfect knowledge

**Figure 2:**
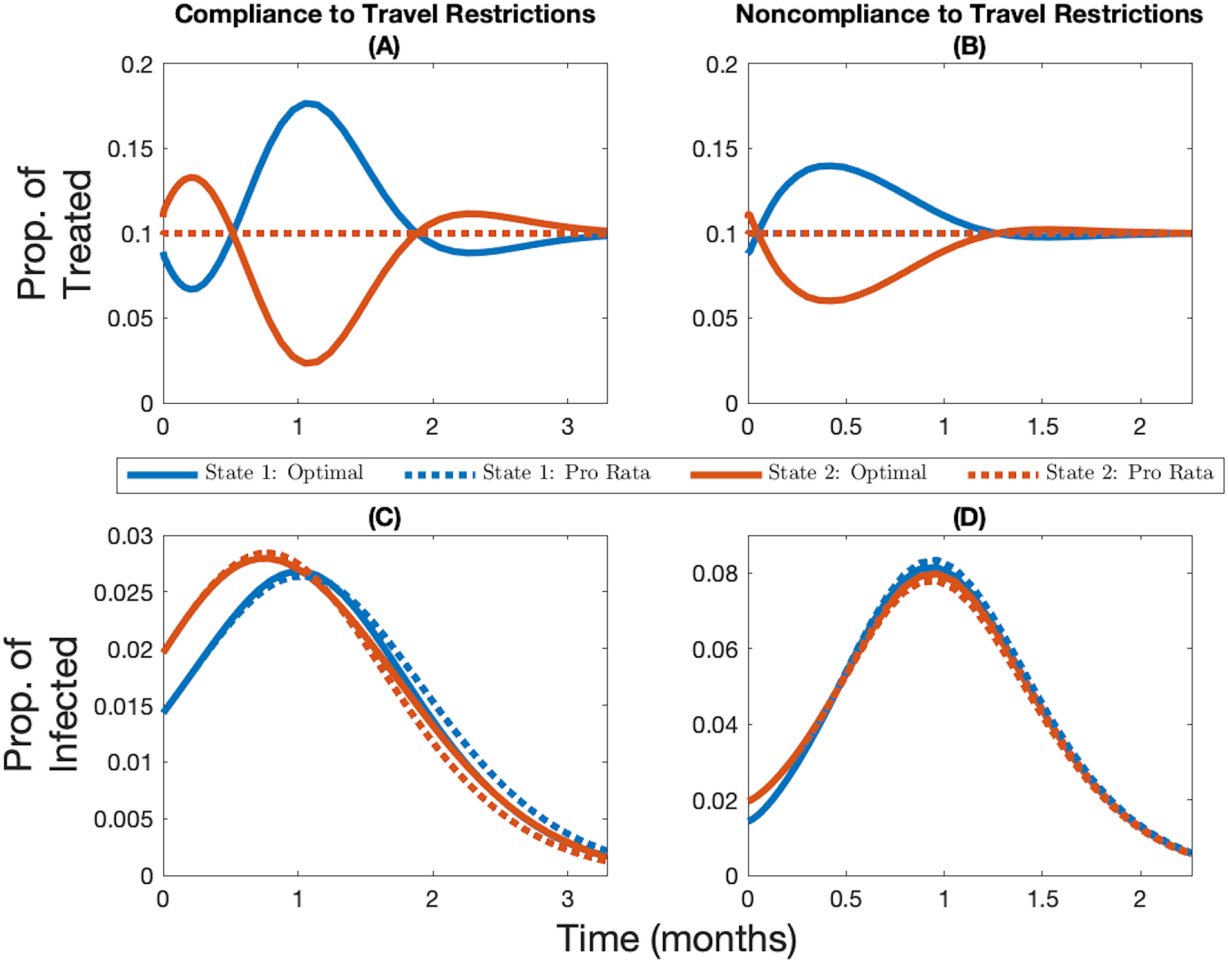
Vaccine allocation with and without compliance to travel restrictions. Change over time in the optimal and pro rata allocations (**A**,**B**) and the corresponding infection levels (**C**,**D**) for State 1 (in blue, the initially lowest-burdened state) and State 2 (in red, the initially highest-burdened state) depending on whether there is compliance to travel restrictions (**A**,**C**) or not (**B**,**D**) for the case where the vaccine capacity constraint is 10% and immunity is permanent. Note the changing *y*-axis in panels (**C**) and (**D**) in order to better highlight the infection levels.

Compared to a pro rata vaccine distribution, the optimal allocation prioritizes the jurisdiction that has the lowest initial infection level (i.e., State 1, in blue, in Fig. 2). However, the amplitude of the optimal deviation and the number of times priority optimally switches from one jurisdiction to another is scenario-specific and depends on travel restrictions (see Fig. 2 and Supplementary Fig. S1), vaccine capacity (see Supplementary Figs. S2, S3, S4, and S5), immunity length (see Supplementary Figs. S6 and S7), and demographic characteristics (see Supplementary Figs. S11, S12, S13, and S14). Similar results on the optimal switch point were found in Ndeffo Mbah and Gilligan^19^.

Population movement from one jurisdiction to another (i.e., when the policymaker observes noncompliance to travel restrictions, or when none are imposed) decreases the structural heterogeneity in the system. As a result, the pro rata rule performs relatively better when populations mix with each other (see Fig. 2 for when immunity is permanent and see Supplementary Fig. S1 for when immunity is temporary), although the mixing of population has a negative health impact because more contacts occur on average.

Regardless of whether or not populations mix, and regardless of whether immunity is temporary of permanent, a higher vaccine capacity implies a relatively smaller deviation from the pro rata rule, meaning that a higher vaccine supply increases the relative performance of the pro rata rule (see Supplementary Figs. S2 and S3 for the case where immunity is permanent; see Supplementary Figs. S4 and S5 for the case where immunity is temporary).

Interestingly, how temporary immunity affects the relative performance of the pro rata rule depends on whether or not the populations from the jurisdictions interact. When populations do not mix (i.e., comply with travel restrictions), temporary immunity has little effect on its performance (see Supplementary Fig. S6; it slightly increases back-and-forth movement of resources between jurisdictions), but when populations mix (i.e., do not comply with travel restrictions, or when none are imposed), it further dampens the structural heterogeneity in the system since the infection and recovery level of both jurisdictions will eventually reach the same positive steady-state level (recall the only heterogeneity in the system is the initial disease burden in the base case; see Supplementary Fig. S7). In the latter case, temporary immunity implies the pro rata rule performs relatively better.

While by definition the pro rata rule we use prioritizes equity of distribution, the optimal allocation of vaccine we derive as a benchmark is unequal from a resource allocation perspective. Instead, it tends to equalize the current infection levels across jurisdictions which means that it prioritizes equity of outcomes. As a result, these optimal cost-minimizing deviations from the pro rata rule lead to more inequality in the cumulative infection level while the pro rata rule leads to more equality in the cumulative infection level (see Supplementary Figs. S8, S9, and S10 for when the vaccine capacity is 5%, 10%, and 15%, respectively).

Introducing heterogeneity in the demographic characteristics of the jurisdictions also impact the relative performance of the pro rata rule. If some jurisdiction has an older population on average, we expect SARS-Cov-2 to have a higher case-fatality ratio in that jurisdiction^14^. These differences in the age structure lead the optimal allocation to favor even more the least infected jurisdiction, which is also the most vulnerable (i.e., with more older individuals) of the two populations because the benefits of vaccination are no longer homogeneous across jurisdictions (see Supplementary Fig. S11 for when immunity is permanent and see Supplementary Fig. S12 for when immunity lasts 6 months). When one jurisdiction has more essential workers than the other (for more details on how the risk of infection is occupation-dependent, see^15^), priority is given to the jurisdiction with a higher contact rate (i.e., with more essential workers) in almost all cases (see Supplementary Fig. S13 for when immunity permanent and see Supplementary Fig. S14 for when immunity lasts 6 months). Overall, introducing heterogeneity in the case fatality ratio means targeting the most vulnerable (i.e., older individuals, or more essential workers) jurisdiction is preferable, and as such, these sources of heterogeneity weaken the relative performance of the pro rata rule. On the other hand, when there is minimal heterogeneity in the age structure and number of essential workers across jurisdictions, the pro rata distribution performs relatively better.

### When decisions must be made without perfect knowledge

**Figure 3:**
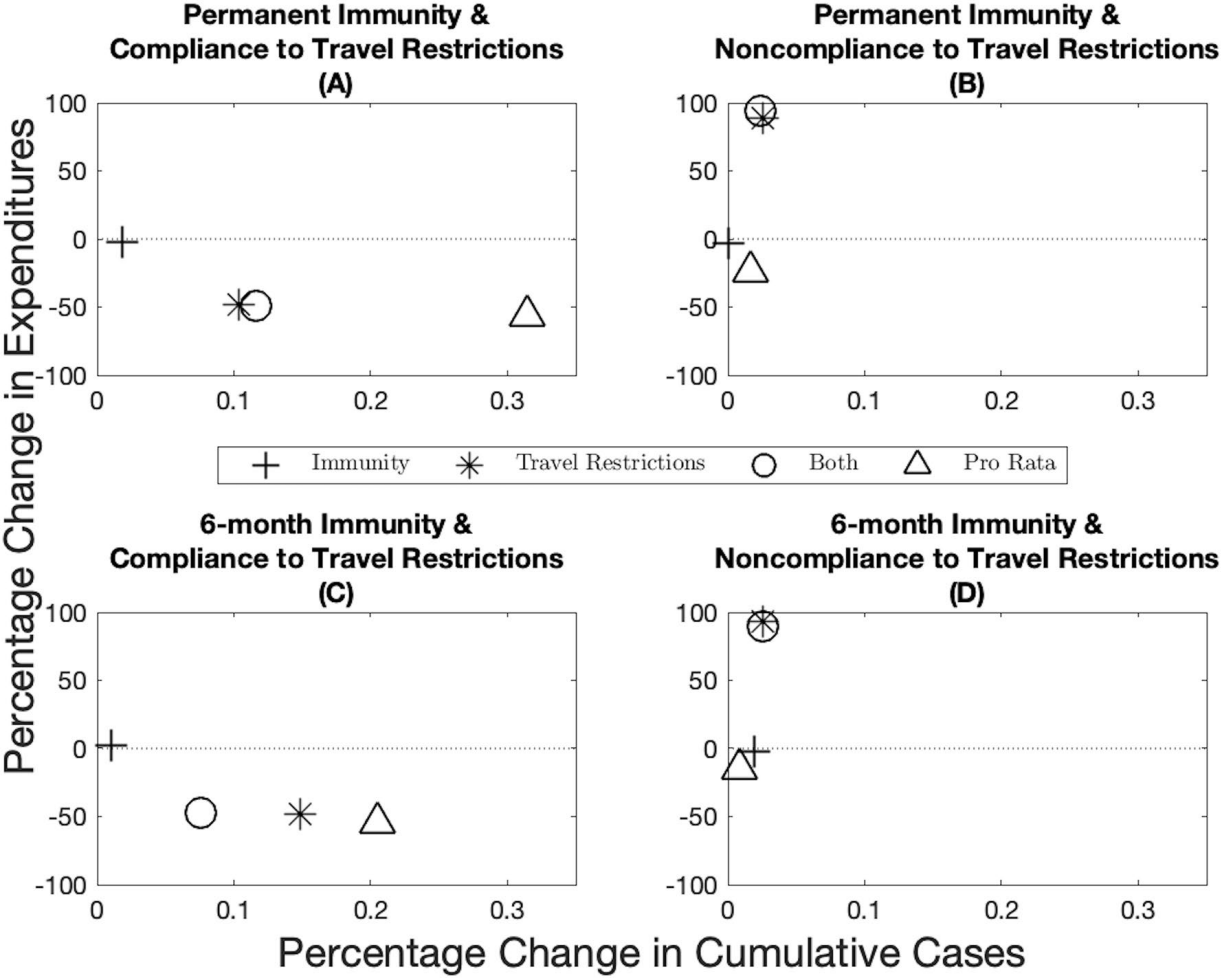
Robustness of epidemiological and economic outcomes under different scenarios when the source of heterogeneity is the timing of the outbreak (the outbreak started earlier in one jurisdiction). Percentage change in expenditures (*y*-axis) and percentage change in cumulative cases (*x*-axis) from the optimal allocation for different immunity-travel restrictions scenarios and for when vaccine capacity is 10%. The *x*-axis represent small percentage changes but when scaled up to population level effects translate into significant differences in public health outcomes.

There is significant uncertainty associated with the duration of immunity (i.e., if it is permanent or temporary) and to what extent populations comply with travel restrictions. One argument for the pro rata rule is that uncertainty in these parameters makes the optimal allocation impossible to achieve. This uncertainty is not yet resolved and public health officials have to choose vaccine allocations based on potentially incorrect assumptions. We compare the robustness of the optimal spatial allocation to the pro rata rule. By definition, the optimal allocation minimizes the net present value of the health-related damages and total expenditures (including vaccine expenditures and the workability cost incurred because of the deviations from the pro rata rule), and thus when it is based on correct assumptions, it cannot do worse on this dimension than the pro rata rule. We measure robustness by first inserting the optimal solution under one set of assumptions into the disease dynamics under another set and compute the changes in total expenditures (i.e., the pharmaceutical intervention and the workability cost) and public health outcomes (cumulative cases) over time. We then calculate the distance of these changes in percentage terms to the optimal solution derived under the “correct” assumptions (represented by the point (0, 0) in Fig. 3). For example, suppose immunity is permanent and there is perfect compliance to a travel restriction (Fig. 3A). We derive the optimal policy under these assumptions and use it to measure the robustness of the optimal policies that are derived under assumptions that immunity is temporary and/or there is noncompliance. The pro rata rule being based on observable factors is then compared to the incorrectly applied optimal policies. Continuing with the above example where immunity is permanent and there is perfect compliance to a travel restriction (Fig. 3A), if the optimal allocation is derived under the assumption that there is permanent immunity but noncompliance to a travel restriction (star marker in Fig. 3A), then we observe approximately 0.1% increase in the cumulative number of cases across jurisdictions and a 50% decrease in cumulative expenditures. We illustrate the case for 10% scarcity below and include other scarcity cases in Appendix C.

Despite the fact that public health practitioners may be required to make allocation decisions based on incomplete information and that this diminishes the performance of the optimal allocation, the optimal allocation still outperforms the pro rata rule in most cases. When demographic characteristics are homogeneous across jurisdictions, we find overall that immunity length has a lesser impact on both economic and epidemiological outcomes than compliance to travel restrictions (compare the distance from the origin between the plusses and the stars in Fig. 3). Importantly, when there is compliance to travel restrictions, the pro rata rule performs worse than any of the optimal allocations while it performs relatively well when there is no compliance to travel restrictions. Across the economic dimension (expenditures), for example, we find that assuming compliance when in fact there is very little leads to greater expenditures (recall that, by design, the pro rata rule has lower expenditures than the optimal policies because the central planner is not incurring the workability costs from deviating off of the allocation). At the same time, we see greater cumulative cases when the opposite holds, that is, assuming no compliance when in fact there is compliance. There are more nuanced trade-offs, however (e.g., compare position of the stars across the panels in Fig. 3), and in some instances the combined effect of incorrectly assuming the wrong immunity and compliance can offset some deviations (e.g., see Fig. 3C) while in other cases the results are dominated by noncompliance to travel restrictions (e.g., see Fig. 3A). Varying the level of scarcity does not change the qualitative nature of results (see Supplementary Fig. S15 and S16 for when vaccine capacity is 5% and 15% respectively), except for one anomaly where the pro rata rule does not always perform worse under assumptions on compliance to travel restrictions (Supplementary Fig. S16).

**Figure 4:**
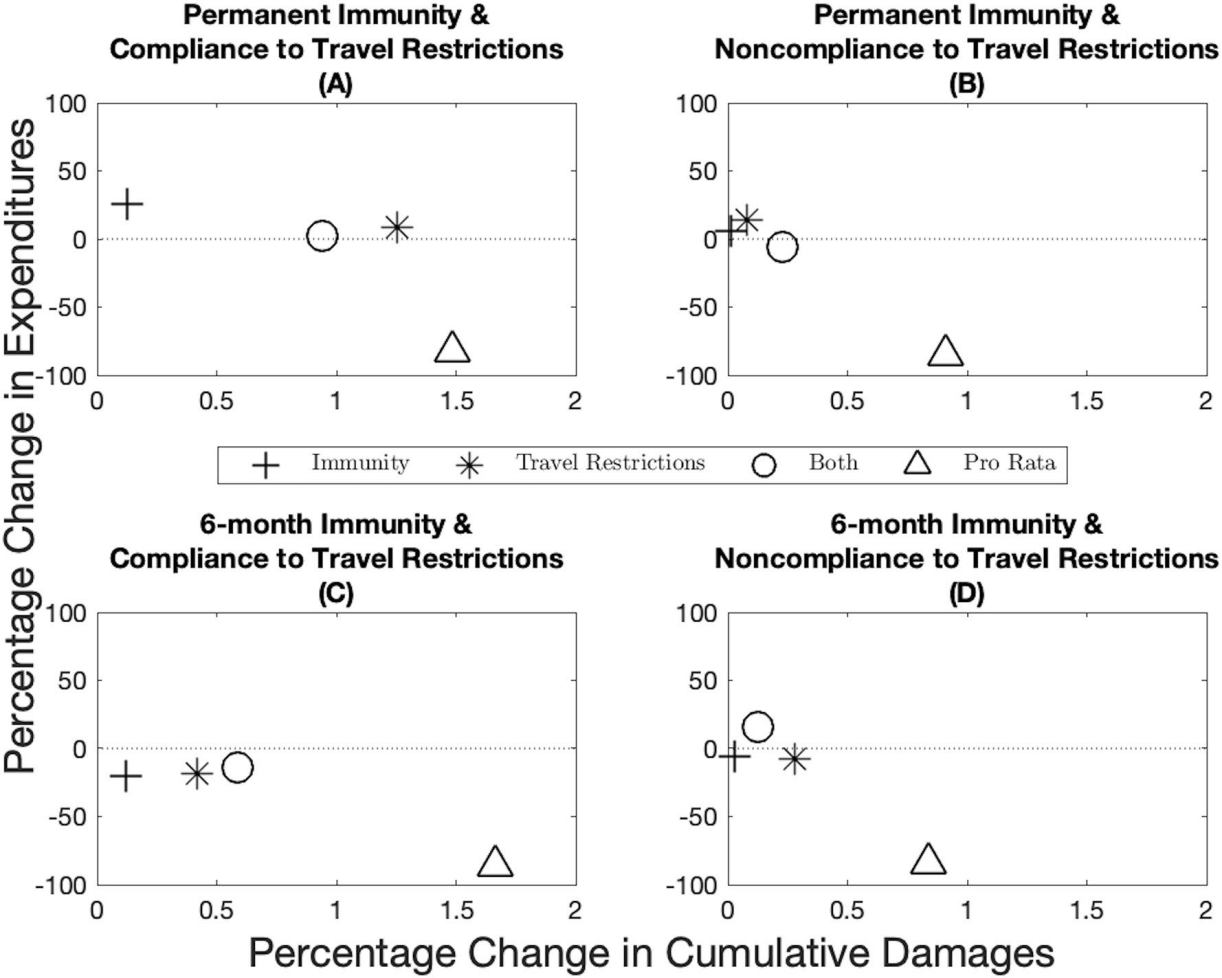
Robustness of epidemiological and economic outcomes under different scenarios when the source of heterogeneity is the case-fatality ratio. Percentage change in expenditures (*y*-axis) and percentage change in cumulative damages (*x*-axis) from the optimal allocation for different immunity-travel restrictions scenarios and for when vaccine capacity is 10%. Note that compared to Supplementary Fig. S17, the use of cumulative damages in this figure gives a more accurate depiction of the situation because cases across jurisdictions are not homogeneous when the case-fatality ratio is different.

We also investigate the robustness of the optimal allocations when the demographic characteristics are heterogeneous across jurisdictions. When jurisdictions have a different case-fatality ratio, the pro rata rule performs better than the optimal allocations when considering cases as the main health outcome (Supplementary Fig. S17). However, this approach is misleading because when case-fatality ratios are heterogeneous across jurisdictions, the cumulative aggregate number of cases (all jurisdictions together) is a poor outcome measure as a case in one place is not equivalent to a case in another jurisdiction. In this setting, the disease burden and cumulative damages give a more accurate depiction of the situation. In fact, while the pro rata rule outperforms the optimal allocations in terms of cumulative cases, it performs considerably poorer when considering cumulative damages. We generally find that the optimal allocations outperform the pro rata rule in all scenarios considered (Fig. 4).

The nuance of focusing on cases or disease burden highlights the importance of decision-makers being specific about their objective: preventing cases, regardless of the severity, or preventing some measure of disease burden? When the objective is the latter, as we assumed in this paper, the simple allocation rule performs considerably poorer because it does not account for prioritization of certain groups of individuals (e.g., older individuals). Demographic heterogeneity has a considerable impact on the optimal vaccine allocation (compare Supplementary Fig. S11 with Fig. 2, and Supplementary Fig. S12 with Supplementary Fig. S1). When comparing Figs. 3 and 4, we can see that introducing heterogeneity in the case-fatality ratio makes the pro rata rule relatively worse than the optimal allocations, even when the optimal allocations are based on incorrect assumptions (this was not always the case in Fig. 3). This highlights that, while in certain cases where population demography is homogeneous across jurisdictions, large heterogeneities in demographic characteristics can really diminish the performance of the pro rata rule and tailored, optimal, allocations can lead to a substantial public health benefit.

**Figure 5:**
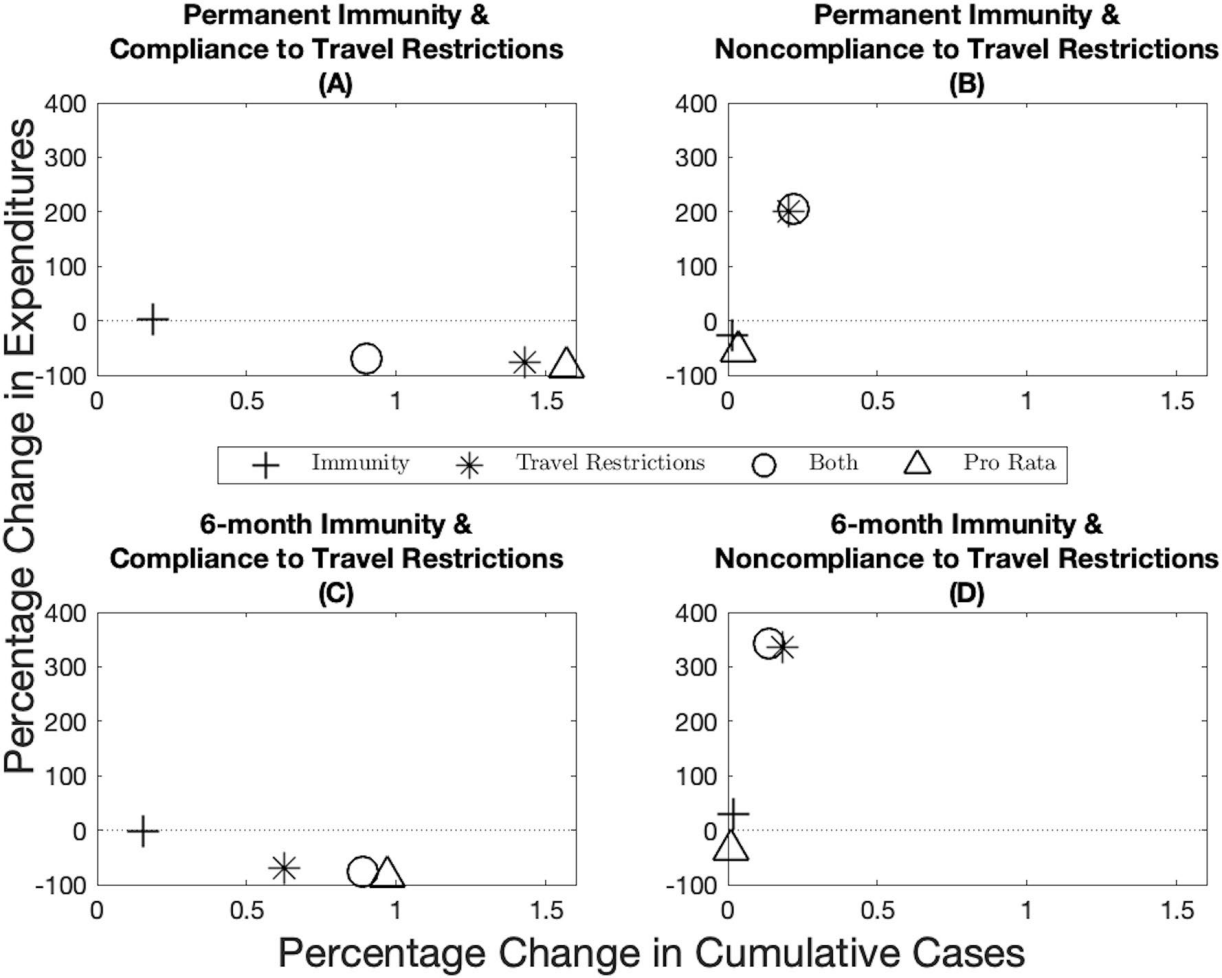
Robustness of epidemiological and economic outcomes under different scenarios when the source of heterogeneity is the contact rate. Percentage change in expenditures (*y*-axis) and percentage change in cumulative cases (*x*-axis) from the optimal allocation for different immunity-travel restrictions scenarios and for when vaccine capacity is 10%.

When jurisdictions have a different contact structure—for instance because one jurisdiction has more essential workers^15^—the same pattern as in Fig. 3 holds in the sense that when there is compliance to travel restrictions, the optimal allocations based on incorrect information outperform the pro rata rule, while the pro rata rule generally performs better than the optimal allocations based on incorrect information when there is noncompliance to travel restrictions (Fig. 5). Compared to the base case of Fig. 3, one important difference to note is the scale of the axes. Across the economic dimension (expenditures), the allocation decision changes considerably depending on whether the jurisdictions comply to travel restrictions or not, which means large variations in workability costs and thus in expenditures (see Supplementary Figs. S13 and S14). Compared to Fig. 3 on the health dimension (cumulative cases), we see that the health consequences of incorrect information is considerably higher when some heterogeneities in the contact structure exist and that the pro rata rule generally performs almost as well as an optimal allocation based on incorrect information.

### Sensitivity analyses

The previous section considers the robustness of optimal allocations to incorrect assumptions about parameters (e.g., assuming permanent immunity while in fact it is temporary). Public health officials will also want to know how much optimal allocations change when parameters change (e.g., because vaccine effectiveness is lower against a new strain of the virus). We address those questions in this section. While both sets of analyses address parameter uncertainty, you can consider in this section that the uncertainty is resolved before the public health officials have to make the vaccine allocation, while in the previous section the uncertainty was not resolved and public health officials had to choose allocations based on potentially incorrect assumptions.

Two key parameters in our analysis are the scale of the workability cost ($c_{A}$ in Eq. (9)) and the level of vaccine effectiveness (see Appendix C for more details). While imposing the pro rata rule ex ante implicitly means that the cost of deviating from it is infinite, in practice it is likely finite but hard to quantify, as it depends on logistical, political, and cultural factors. We investigate the sensitivity of our results by solving for optimal vaccine allocation over a range of values. We find greater deviations off of the pro rata rule at lower workability costs resulting in greater differences in cumulative cases, and smaller deviations as the workability cost parameter increases (Supplementary Fig. S18A–D). Specifically, we find that when the cost is in the neighborhood of the VSL (*c* in Eq. (7) and Supplementary Fig. S18 black line represents the VSL), that the planner no longer deviates from the pro rata rule.

The base case parameter for vaccine effectiveness we utilized in the paper is based on estimates of the influenza vaccine^43^ (see Appendix A for more details) and represents a conservative estimate similar to the lower efficacy vaccines listed for emergency use listing by WHO (i.e., the Sinopharm COVID-19 vaccine, see for instance^9^). Evidence from other COVID-19 vaccines (e.g., Pfizer/BioNTech and Moderna vaccines) suggest that effectiveness could be considerably higher than our base case scenario^10^, but evidence suggests that vaccine efficacy is lower against new variants (e.g., the Delta^11^ and Omicron^12^ variants). In addition to the above immunological motivations for this sensitivity analysis, there is also an important modeling motivation for it. In this paper, we made the simplifying assumption that only susceptible individuals can receive a vaccine, while in practice this is clearly not the case. Incorporating this change to our model would mean that only a fraction of all available vaccines would effectively work at reducing the susceptible class, which essentially translates to a reduction of the vaccine effectiveness. When varying the effectiveness of the vaccine, we find that the more effective a vaccine is, the more a central planner would want to deviate from the pro rata rule (in blue; Supplementary Fig. S19A–D). As a result of this greater deviation, we see a larger difference in terms of the reduction in cumulative cases (in red; Supplementary Fig. S19A–D).

## Discussion

Recent studies have discussed how a vaccine against the coronavirus disease (COVID-19) should be allocated within a geographical area (see for instance^1–3^) and on a global scale (see for instance^4–6^). Building off the spatial-dynamic literature in epidemiology, we contribute to this body of work by addressing the question of distributing a scarce allotment of COVID-19 vaccine across smaller geographic areas, such as counties or states, and by showing how a pro rata vaccine distribution rule that favors “speed and workability” (put forward by the National Academies of Sciences, Engineering, and Medicine (NASEM)^7^, and the World Health Organization (WHO) has a similar principle^8^) performs compared to an optimal allocation when allocation decisions must be determined before uncertainty about key behavioral (i.e., compliance to travel restrictions) and epidemiological (i.e., the length of immunity to the disease) factors are resolved. Countries receiving allotments of vaccines via COVAX—a WHO led initiative aimed to provide an equitable access to COVID-19 vaccines—could follow NASEM’s^7^ and WHO’s^8^ principle and allocate a COVID-19 vaccine to jurisdictions within their borders based on the jurisdictions’ population size. This approach, which prioritizes equity of distribution, is a better approximation of the optimal allocation, which is more closely aligned with equity of outcomes, when immunity is shorter, when populations from different jurisdictions mix with each other, when vaccine supply is high, and when demographic characteristics are similar across jurisdictions. Despite potential economic and public health benefits of deviating from this prevailing pro rata rule, the uncertainty around behavioral and epidemiological factors needed to determine the optimal allocation diminishes the feasibility, and potentially the performance, of the optimal allocation. While there are many factors that come into play in these allocation decisions, the methodology proposed here provides a way to benchmark these rules to illustrate the trade-offs. Other methodologies, that do not solve for the optimal policy, are left to benchmark the pro rata rule against another rule of thumb, where the set of possible rules of thumb is infinite.

We considered several different scenarios where the length of immunity, the compliance to travel restrictions, the size of the vaccine allotment, and the demographics across jurisdictions are varied. In most of these scenarios, we find that priority should be given to jurisdictions that initially have lower disease burden (i.e., lower infection level). The intuition behind this result—already put forward by Rowthorn et al.^18^ when investigating optimal control of epidemics in a scenario where no immunity to the disease is developed—is that the priority should be to protect the greater population of susceptible individuals, and that focusing on a subset of the population, rather than on the entire population, can make a significant difference^44^. However, prioritizing the jurisdiction with a lower disease burden implies some workability costs^7^, i.e., social costs due to logistical, political, or cultural factors that are incurred for deviating from the status quo; the larger these costs are, the closer the pro rata rule is to the optimal allocation. Future research considering nonlinear damages due to an overload of health care systems^45^ and a corresponding varying death rate due to scarce intensive care unit beds^23^, and other second-order problems such as consumption losses^46,47^, excess mortality^48^, and psychological distress^38^ would be necessary to further assess the relative performance of the pro rata rule.

While other rules of thumb may be preferable to the pro rata rule put forward by NASEM^7^ and WHO^8^, fully assessing their effectiveness is challenging as it is not immediately clear what the workability costs avoided from adopting these alternative rules would be (i.e., it would not be zero as they imply a deviation from the status quo). Future work to address the effectiveness of another rule of thumb relative to the optimal allocation could use the optimal control methodology employed in this paper to offer important information to policymakers that face the challenge of allocating scarce life-saving resources to their jurisdictions.

There are other important factors that have received significant attention in the literature that could be the focus of future research. For example, we assumed that jurisdictions have the same vaccine distribution capabilities, while in practice it is likely that jurisdictions will differ in this aspect for various reasons such as vaccine hesitancy^49^ and pre-allotment preparedness^50^. Our model further assumes that decision-makers have complete knowledge about the number of infected individuals in both patches, yet challenges with reporting is common for infectious diseases^51^, including COVID-19^52–54^. We have also assumed that individuals do not change their behavior following vaccination, though individuals may engage in riskier behavior^55^ or behavioral intervention policies may be lifted^56^. This effectively creates a trade-off between the vaccination level and the contact rate. We have also simplified the cost structure of the model by only considering the economic health costs stemming from the infection rate; the cost of shutting down the economy^39^ may impact vaccine allocation decisions when economic impacts are heterogeneous across jurisdictions. We simplified vaccine dosage—for instance, whether to delay or not the second dose^57^ and whether or not to use fractional dosing^58^. We have also simplified vaccination by assuming vaccine failure was readily observable and those individuals could be revaccinated. While in practice individuals with vaccine failure would remain susceptible. We leave for future work an investigation of the case where the number of individuals with vaccine failure is sufficiently large. Finally, we have only considered uncertainty that is either resolved before the allocation decision or never resolved at all; another possibility is that more information about parameters is learned during the allocation decision (see, for instance^59^, where cell phone data gradually informs mobility patterns for COVID-19). In such cases, adaptive management approaches may be of relevance^60^. Further research including these aspects could add additional valuable insights into the trade-offs inherent in these different allocations rules.

Finally, while our paper and most of the discussion revolves around the allocation of a vaccine, a similar allocation problem has arisen with antiviral drug becoming available (for a discussion on antiviral treatments for SARS-Cov-2, see^61^). Because drugs and vaccines have different goals—treating infected individuals and prophylaxis, respectively—the economic and public health trade-offs of different allocation rules may be unique to the type of pharmaceutical intervention. Future work considering the joint allocation question of antiviral drugs and vaccines could be valuable in understanding the trade-offs and complementarities between these different pharmaceutical interventions.

## Supplementary Information

Supplementary Information.

## Data Availability

All data needed to evaluate the conclusions in the paper are present in the paper and Supplementary Materials. The data and Matlab/TOMLAB code used to set-up the model and run the numerical analyses is available on Zenodo at https://doi.org/10.5281/zenodo.6412668 and on Github at https://github.com/fmcastonguay/SpatialAllocationCOVID19.
