# Supplementary material for: Optimal spatial evaluation of a pro rata vaccine distribution rule for COVID-19
Source: Sci Rep. 2023 Feb 7;13:2194. doi: 10.1038/s41598-023-28697-8 (PMC9904532; doi:10.1038/s41598-023-28697-8)
Supplement: Supplementary file 1 — Supplementary Information. [file 41598_2023_28697_MOESM1_ESM.pdf]

Supplementary Material

Optimal Spatial Evaluation of a Pro Rata Vaccine Distribution Rule for COVID-19

GitHub repository:
https://github.com/fmcastonguay/SpatialAllocationCOVID19

François M. Castonguay,[1*] Julie C. Blackwood,[2] Emily Howerton,[3]
Katriona Shea,[3] Charles Sims,[4] and James N. Sanchirico[5,6]

[1]Department of Agricultural and Resource Economics,
University of California, Davis, Davis, CA 95616, USA
[2]Department of Mathematics and Statistics, Williams College, Williamstown, MA 01267, USA
[3]Department of Biology and Center for Infectious Disease Dynamics,
Pennsylvania State University, University Park, PA 16802, USA
[4] Howard H. Baker Jr. Center for Public Policy and Department of Economics,
University of Tennessee, Knoxville, Knoxville, TN 37996, USA
[5]Department of Environmental Science and Policy,
University of California, Davis, Davis, CA 95616, USA
[6]Resources for the Future, Washington DC 20036, USA

[*]To whom correspondence should be addressed; E-mail: fcastonguay@ucdavis.edu

Appendices

Contents

A Parameterization **2**
A.1 Epidemiological Model . . . 2
A.2 Economic Model . . . 2
A.3 Parameter Levels . . . 4

B Optimization **5**
B.1 Boundary Conditions . . . 5
B.2 Nonnegativity and Upper-Bound Constraints . . . 5
B.3 Capacity Constraints of the Pharmaceutical Interventions . . . 6
B.4 Numerical Methods . . . 6

C Figures **7**
C.1 Homogeneous Demographic Characteristics . . . 7
C.1.1 Compliance and Noncompliance to the Travel Restrictions . . . 7
C.1.2 Vaccine Capacity Constraints when Immunity is Permanent . . . 8
C.1.3 Vaccine Capacity Constraints when Immunity is Temporary . . . 9

C.1.4 Permanent vs Temporary Immunity . . . 10
C.1.5 Cumulative Infection Levels . . . 11
C.2 Heterogeneous Demographic Characteristics . . . 13
C.2.1 Heterogeneous Case-Fatality Ratio . . . 13
C.2.2 Heterogeneous Contact Rate . . . 14
C.3 Robustness of Spatial allocations . . . 15
C.3.1 Homogeneous Demographic Characteristics . . . 15
C.3.2 Heterogeneous Case-Fatality Ratio . . . 16
C.4 Sensitivity Analyses . . . 17
C.4.1 Workability Cost . . . 17
C.4.2 Vaccine Effectiveness . . . 19

A Parameterization

A.1 Epidemiological Model

According to Diekmann et al. (62), the basic reproduction ratio R_0 of any disease is given by the expected number of secondary infection caused a by a typical infected individual over its entire infectious period, at a disease-free equilibrium. In the most basic epidemiological model, the R_0 is simply given by the contact rate multiplied by the mean infectious period. When considering more complex models—as the two-jurisdiction SEIR model in this paper—one needs to use the next-generation matrix and find its dominant eigenvalue to find the R_0 (62). Denote two matrices by F and V, and let the ij^{th} element in F represents the rate at which infected individuals in population j produce new infections in population i, and the ij^{th} element in V represents the transition rate between $(i \neq j)$, or out of $(i = j)$, infectious compartments; the next-generation matrix is equal to $-FV^{-1}$. In the model presented in this paper,

$$F = \begin{pmatrix} 0 & \beta_{11} & 0 & \beta_{12} \\ 0 & 0 & 0 & 0 \\ 0 & \beta_{21} & 0 & \beta_{22} \\ 0 & 0 & 0 & 0 \end{pmatrix} \text{ and } V = \begin{pmatrix} -\sigma & 0 & 0 & 0 \\ \sigma & -(\gamma+\varphi_1) & 0 & 0 \\ 0 & 0 & -\sigma & 0 \\ 0 & 0 & \sigma & -(\gamma+\varphi_2) \end{pmatrix}$$

where the four rows of F and V refer to the E_1, I_1, E_2 and I_2 equations, respectively. Note that both matrices F and V are derived under the assumption of introducing a single exposed individuals in an otherwise susceptible population. Given we assume that $\beta_{11} = \beta_{22} = \beta_{ii}$ and $\beta_{12} = \beta_{21} = \beta_{ij}$, and when in our main analysis we let $\varphi_1 = \varphi_2 = \varphi$, the basic reproduction ratio of our model simplifies to,

$$R_0 = \frac{\beta_{ii} + \beta_{ij}}{\gamma + \varphi}$$

for $i = 1, 2$, $j = 1, 2$, and $i \neq j$. We set the basic reproduction ratio $R_0 = 1.43$, according to estimates of the R_0 from Li et al. (63) and using estimates of the effect of nonpharmaceutical interventions on the R_0 from Tian et al. (64). We assume a mean recovery period $(\frac{1}{\gamma})$ of 5 days (65), and a case-fatality ratio of 1.78% (adjusted for misreporting, see (66)) to calibrate the rate of disease induced mortality, φ. Parameters β_{ii} and β_{ij} are then calibrated assuming what Tian et al. (64) call a "medium effect of the [nonpharmaceutical] control" when there is compliance to travel restrictions, and a "lower effect of the [nonpharmaceutical] control" when there is no compliance to travel restrictions; this yields $R_0 \approx 1.4$ with compliance to travel restrictions, and $R_0 \approx 2.1$ when there is no compliance to travel restrictions. The mean latency period $(\frac{1}{\sigma})$, which one needs to know to calculate matrix V even though it does not appear in the basic reproduction ratio, is assumed to last 3 days (65).

A.2 Economic Model

To quantify damages, we use the value of statistical life recommended by the U.S. Environmental Protection Agency (35). The disability weight associated with COVID-19 infection is assumed to be equivalent to a lower respiratory tract infection, which is a value of $w = 0.133$. According to the World Health Organization (WHO), a disability weight is "a weight factor that reflects the severity of the disease on a scale from 0 (perfect health) to 1 (equivalent to death)"; see `https://www.who.int/healthinfo/global_burden_disease/daly_disability_weight/en/` for more details. For more details on how COVID-19's disability resembles lower respiratory tract infections, see (36).

This disability weight thus allows for a comparison between the individuals that are infected with the disease but do not die, and the individuals that die from its complications.

Expenditures related to the pharmaceutical intervention are based off estimates of vaccine costs. Numerous governments around the world, including the U.S. federal government, have contracted biotech companies producing COVID-19 vaccines; governments pay money in exchange of a guaranteed number of doses of COVID-19 vaccines. These estimates and the prices of current influenza vaccine turn out to be approximately 20 U.S. dollars per dose, with two doses per individual; this is the value we chose in our analysis. For COVID-19 vaccine prices, see: https://www.npr.org/sections/health-shots/2020/08/06/899869278/prices-for-covid-19-vaccines-are-starting-to-come-into-focus. For a comparison with influenza vaccine prices, see https://www.cdc.gov/vaccines/programs/vfc/awardees/vaccine-management/price-list/index.html.

The value of the workability cost—inspired by the paper of Ryan et al. (40) where the authors show the implications of policy adjustment costs for fisheries management—is based on a certain proportion of the value of statistical life; in the base case, we assume it to be 3 orders of magnitude smaller. All costs in the model are assumed to be discounted at a 1.5% annual rate (see (67) for a discussion about discounting health-related expenditures).

A.3 Parameter Levels

Table S1 below summarizes the main set of parameter values we used in the numerical simulation.

Parameters	Level	Definition
β_{ii}	8.86	Transmission rate within a given state (month^{-1}).[1]
β_{ij}	4.36	Transmission rate across states (month^{-1}).[1]
σ	10.14	Rate at which infected individuals become infectious (month^{-1}).[2]
γ	6.08	Rate of recovery (month^{-1}).[2]
ω	0.17	Rate at which immunity is lost (month^{-1}).[3]
φ	0.11	Rate of disease induced mortality (month^{-1}).[4]
w	0.13	Disability weight associated with the disease (unitless).[5]
q_V	0.65	Effectiveness of vaccines (proportion).[6]
r	0.0013	Discount rate (month^{-1}).[7]
c_V	40	Cost of treating one individual via vaccine (US Dollars).[8]
c_A	10×10^3	Workability cost (US Dollars).[9]
c	10×10^6	Value of statistical life (US Dollars).[10]

Table S1: **Parameter levels used in the numerical simulation.**

1. β_{ii} and β_{ij} were calibrated using a R_0 estimate from Li et al. (63) and estimates of effects of nonpharmaceutical interventions from Tian et al. (64); this yields a R_0 of approximately 1.4 when there is compliance to travel restrictions and a R_0 of approximately 2.1 when there is no compliance to travel restrictions. These two values represent, respectively, a "medium" and "low" effect of nonpharmaceutical interventions.
2. σ and γ are based on estimates from Davies et al. (65), which represent a 3-day latency period and a 5-day recovery period, respectively.
3. ω represents a 6-month immunity period in the scenarios where we assume immunity is not permanent; based on Edridge et al. (27).
4. φ is calibrated by using a case-fatility rate of 1.78%, and is adjusted for mis- and under-reporting (66).
5. w represents the disability associated with severe lower respiratory tract infections (36).
6. q_V represents a conservative estimate based on the effectiveness of the influenza vaccine (43). This is similar to the lower-bound effectiveness of vaccines listed for emergency use listing by WHO (i.e., the Sinopharm COVID-19 vaccine (9)) and accounts for potential reduced effectiveness of vaccines when faced with emerging variants of concern (11, 12).
7. r is based on results from John et al. (67) that suggest a yearly discount rate between 0.3% and 1.5% for health related expenditures. We chose a 1.5% annual discount rate in the main set of results which gives a monthly discount rate of $r = 0.0013$.
8. c_V is based on the assumption that an individual requires two doses and is based on initial agreements between the U.S. federal government and biotech companies; see `https://www.npr.org/sections/health-shots/2020/08/06/899869278/prices-for-covid-19-vaccines-are-starting-to-come-into-focus`. For a list of current vaccine prices, and particularly the price of the influenza vaccine, see `https://www.cdc.gov/vaccines/programs/vfc/awardees/vaccine-management/price-list/index.html`.
9. c_A is based on a certain proportion of the value of statistical life, c (defined below); in the base case, we assume it is 3 orders of magnitude smaller.
10. c represents a value of statistical life of 10M U.S. dollars, which is roughly based on the value of a statistical life that the U.S. Environmental Protection Agency (35) uses: approximately $7.4 million ($2006) which is equivalent to approximately $9.54 million ($2020).

Table S2 below summarizes the differences in parameter values we used in the numerical simulation when the source of heterogeneity across jurisdiction is varied.

Parameters	Source of the Heterogeneity Across Jurisdictions		
	**Timing of the Outbreak**	**Case-Fatality Ratio**[1]	**Contact rate**
β_{11}	8.86	8.95	8.45
β_{22}	8.86	8.86	9.27
β_{12}	4.36	4.41	4.77
β_{21}	4.36	4.36	3.95
φ_1	0.11	0.17	0.11
φ_2	0.11	0.11	0.11

Table S2: **Difference in parameter levels when source of heterogeneity is varied.**

1. Note that because the contact rates β_{ii} and β_{ij} are dependent on the case-fatality ratio φ_i—as they are clalibrated to match a certain value of the basic reproduction number R_0—the values for β_{ii} and β_{ij} also change when φ_i changes. Note that the converse is not true.

B Optimization

B.1 Boundary Conditions

To yield the initial conditions of the optimal control problem, we calibrated the model using the above parameter values and simulated out a COVID-19 outbreak in two identical jurisdictions, where we assumed there was one exposed individual in an otherwise entirely susceptible population of 10 million individuals. We assumed that both jurisdictions undertook nonpharmaceutical interventions that had a "medium effect" on the basic reproduction ratio (64) (i.e., that there was perfect compliance to travel restrictions). After simulating out the disease dynamics for a period of eight months and two weeks, and eight months and three weeks for Jurisdiction 1 and Jurisdiction 2 respectively, the initial conditions yield were:

**Jurisdiction**	N_i	S_i	E_i	I_i	R_i
Jurisdiction 1	1	0.9074	0.0103	0.0143	0.0667
Jurisdiction 2	1	0.8662	0.0138	0.0196	0.0986

Table S3: **Initial conditions of the numerical simulation.**

We assume that the terminal conditions (i.e., the conditions on state variables in $t = T$, the final time period) are free to be optimally determined. Formally, the initial and terminal conditions of the ten state variables are such that:

$$S_i(0), E_i(0), I_i(0), R_i(0), \text{ and } N_i(0) \text{ are given for } i = 1, 2; \tag{S1a}$$

$$S_i(T), E_i(T), I_i(T), R_i(T), \text{ and } N_i(T) \text{ are free for } i = 1, 2. \tag{S1b}$$

B.2 Nonnegativity and Upper-Bound Constraints

State variables S_i, E_i, I_i, R_i, and N_i for $i = 1, 2$ are subject to nonnegativity and physical constraints. Formally:

$$0 \le S_i \le N_i \le 1 \text{ for } i = 1, 2; \tag{S2a}$$

$$0 \le E_i \le N_i \le 1 \text{ for } i = 1, 2; \tag{S2b}$$

$$0 \le I_i \le N_i \le 1 \text{ for } i = 1, 2; \tag{S2c}$$

$$0 \le R_i \le N_i \le 1 \text{ for } i = 1, 2; \tag{S2d}$$

$$S_i + E_i + I_i + R_i = N_i \le 1 \text{ for } i = 1, 2. \tag{S2e}$$

Control variables are modelled as direct controls (see examples in (2, 18, 33)) and can be interpreted as a reduction in the number of susceptible individual in a given time period (i.e., a month). Formally, the constraints on the control variables are given by:

$$0 \leq u_{V_i} \leq S_i \text{ for } i = 1, 2. \tag{S3}$$

Because of a limited supply of vaccines (see details below), the physical upper-bound on constraints (S3) will only be binding when capacity constraint is nonbinding. When this occurs, it means that there are fewer susceptible individuals than there are available vaccines.

B.3 Capacity Constraints of the Pharmaceutical Interventions

For completeness, we also include the capacity constraints already mentioned in the main paper. In addition to the physical constraints on the control variables, the aim of our paper is to study how to allocate a limited allotment of vaccines. Hence, the control variables are also subject to

$$u_{V_1} + u_{V_2} \ \leq \bar{u}_V; \tag{S4}$$

when the central planning agency decides to potentially deviate from the pro rata rule. Conversely, the constraints of the pro rata rule are:

$$u_{V_i} \leq \left(\frac{N_i}{N_1 + N_2} \right) \bar{u}_V \quad \text{for } i = 1, 2. \tag{S5}$$

As mentioned in the main paper, the total available quantity of vaccine ($\bar{u}_V$) represents a certain percentage (5%, 10%, or 15%) of the total population size.

B.4 Numerical Methods

Pseudospectral collocation approximates the continuous time optimal control model with a constrained nonlinear programming problem (see (41, 42, 68, 69) for other applications of this technique). The dynamic controls to our problem—i.e., the vaccine allocation—are approximated by a polynomial of degree n (determined by the number of collocation points) over a period from $t = 0$ (date at which the vaccine starts to be administered) to $t = T$ (assumed to be four months after the vaccine administration) (70). The residual error of the constraints is minimized by the algorithm at the n collocation points, where n is chosen to have a reasonable speed of convergence to a solution and a low numerical error. Here, we chose 60 collocations points. In this sort of problem, the main advantage of this approach over more usual methods to solve such two-point boundary problems, such as shooting methods, is that nonnegativity constraints (e.g., on the number of infected individuals) and upper-bound constraints (mimicking e.g., vaccine capacity constraints) on state and control variables can be directly incorporated in the problem (71). This method thus allows us to find optimal solutions that may lay on the boundary of the control set for a certain period of time. For COVID-19 vaccines, this is likely due to the scarcity of the supply of vaccine in the short-term. Another advantage of this method is the ability to deal with large-scale dynamical systems, such as the one presented here with ten state variables and two control variables. The solution was found using TOMLAB (v. 8.4) (72, 73) and the accompanying PROPT toolbox (74). The approximate nonlinear programming problem is solved using general-purpose nonlinear optimization packages (e.g., KNITRO, SNOPT and NPSOL).

C Figures

C.1 Homogeneous Demographic Characteristics

C.1.1 Compliance and Noncompliance to the Travel Restrictions

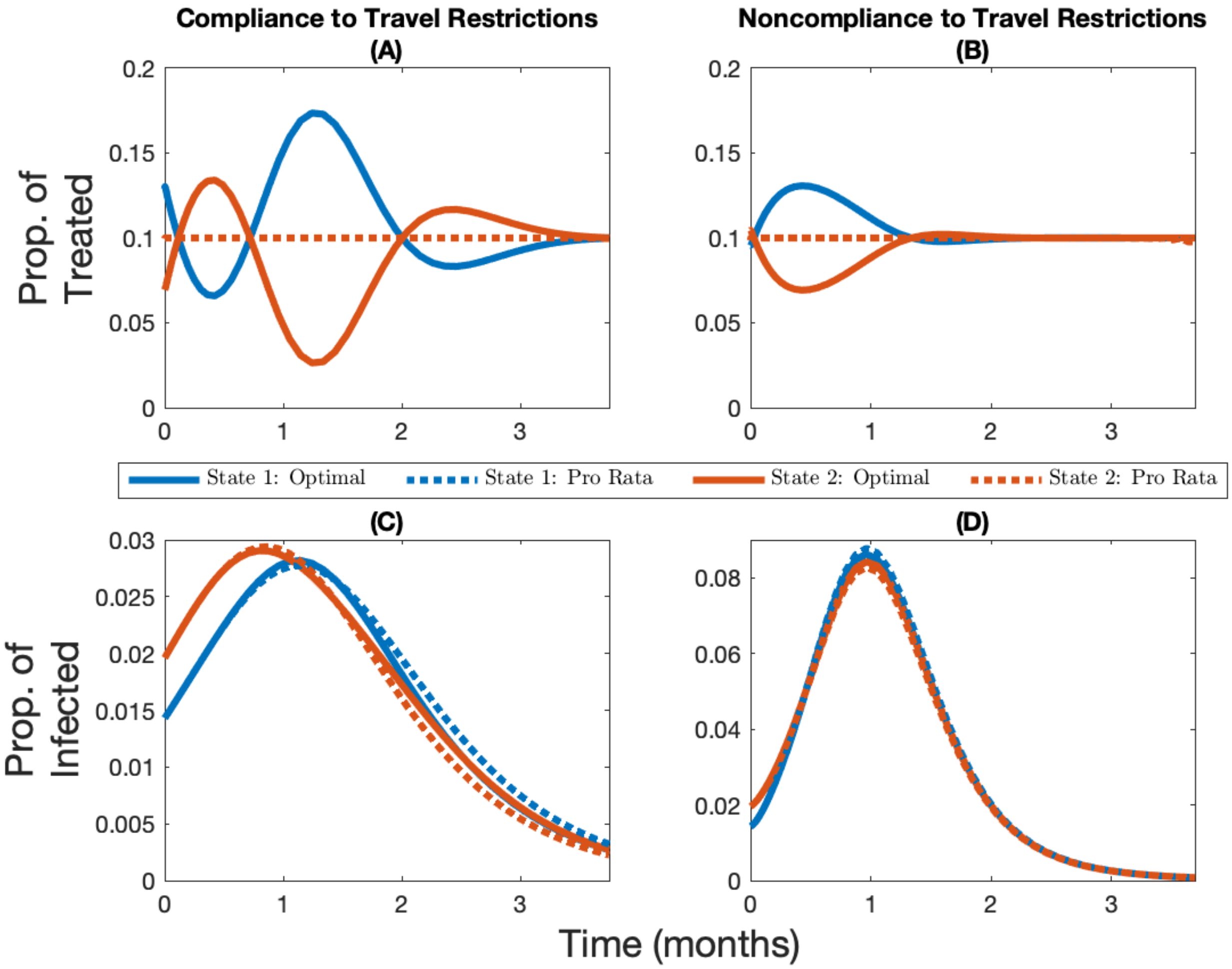

Figure S1: **Permanent immunity with and without compliance to travel restrictions.** Change over time in the optimal and pro rata allocations (panels A and B) and the corresponding infection levels (panels C and D) for State 1 (in blue, the initially lowest-burdened state) and State 2 (in red, the initially highest-burdened state) depending on whether there is compliance to travel restrictions (panels A and C) or not (panels B and D) for the case where the vaccine capacity constraint is 5% and immunity lasts six months.

C.1.2 Vaccine Capacity Constraints when Immunity is Permanent

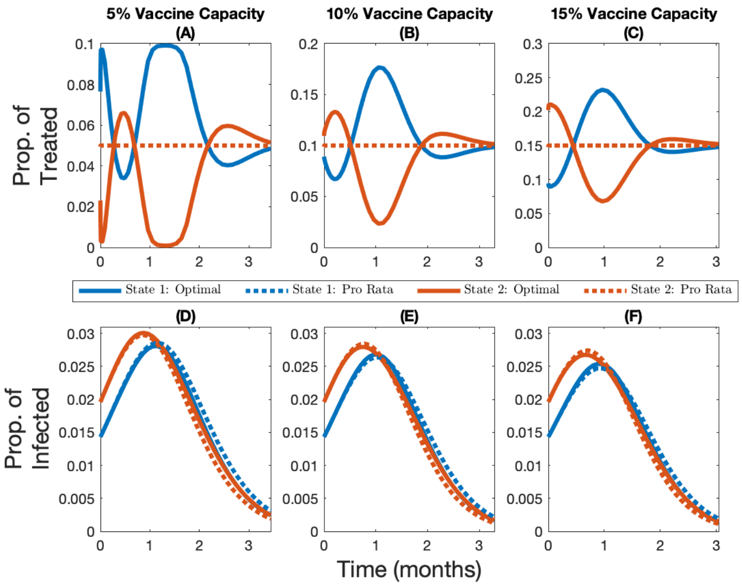

Figure S2: **Permanent immunity and compliance to travel restrictions with 5%, 10%, and 15% vaccine capacity.** Change over time in the optimal and pro rata allocations (panels A, B, and C) and the corresponding infection levels (panels D, E, and F) for State 1 (in blue, the initially lowest-burdened state) and State 2 (in red, the initially highest-burdened state) depending on whether capacity is 5% (panels A and D), 10% (panels B and E), or 15% (panels C and F), for the case where immunity is permanent and there is compliance to travel restrictions.

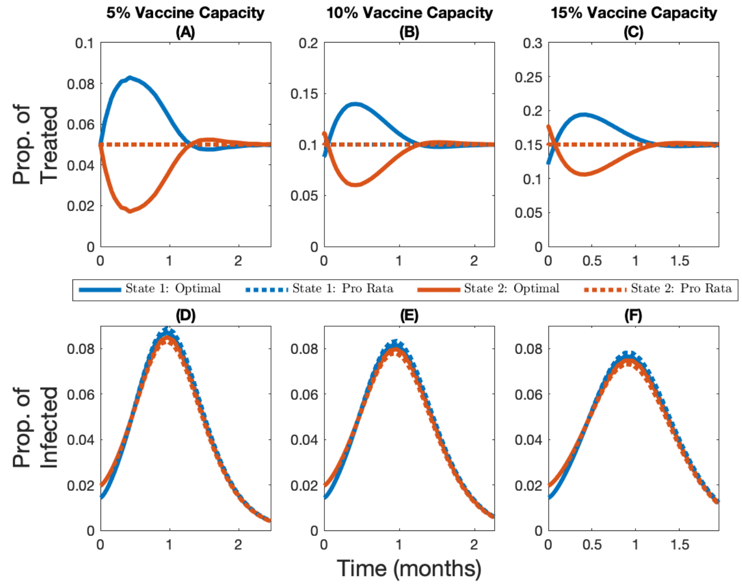

Figure S3: **Vaccine allocations under different levels of scarcity without compliance to travel restrictions**. Change over time in the optimal and pro rata allocations (panels A, B, and C) and the corresponding infection levels (panels D, E, and F) for State 1 (in blue, the initially lowest-burdened state) and State 2 (in red, the initially highest-burdened state) depending on whether capacity is 5% (panels A and D), 10% (panels B and E), or 15% (panels C and F), for the case where immunity is permanent and there is no compliance to travel restrictions.

C.1.3 Vaccine Capacity Constraints when Immunity is Temporary

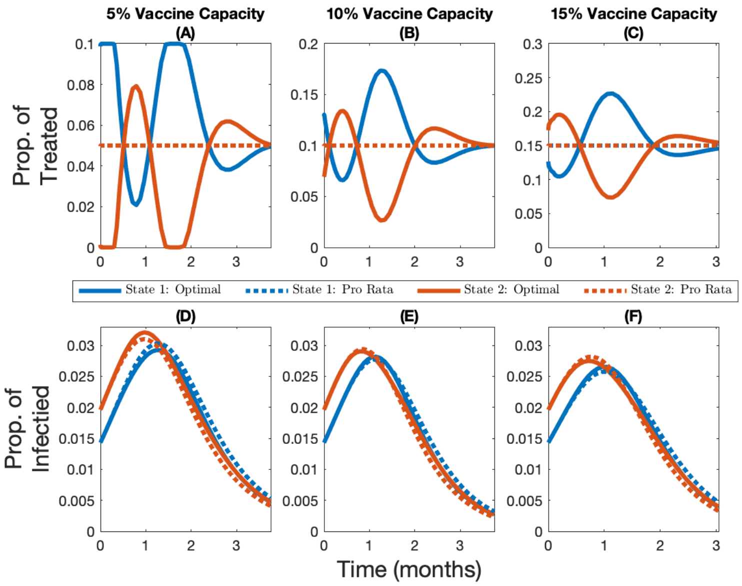

Figure S4: **Temporary immunity and compliance to travel restrictions with 5%, 10%, and 15% vaccine capacity.** Change over time in the optimal and pro rata allocations (panels A, B, and C) and the corresponding infection levels (panels D, E, and F) for State 1 (in blue, the initially lowest-burdened state) and State 2 (in red, the initially highest-burdened state) depending on whether capacity is 5% (panels A and D), 10% (panels B and E), or 15% (panels C and F), for the case where immunity lasts six months and there is compliance to travel restrictions.

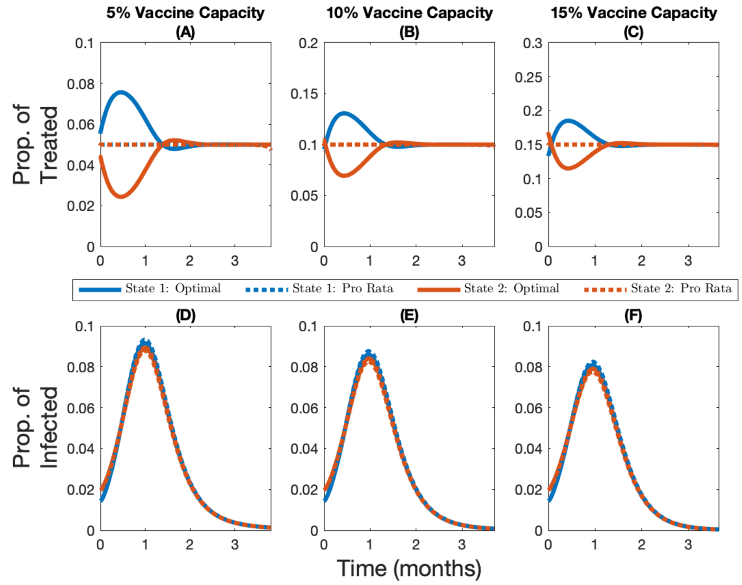

Figure S5: **Temporary immunity and noncompliance to travel restrictions with 5%, 10%, and 15% vaccine capacity.** Change over time in the optimal and pro rata allocations (panels A, B, and C) and the corresponding infection levels (panels D, E, and F) for State 1 (in blue, the initially lowest-burdened state) and State 2 (in red, the initially highest-burdened state) depending on whether capacity is 5% (panels A and D), 10% (panels B and E), 15% (panels C and F), for the case where immunity lasts six months and there is no compliance to travel restrictions.

C.1.4 Permanent vs Temporary Immunity

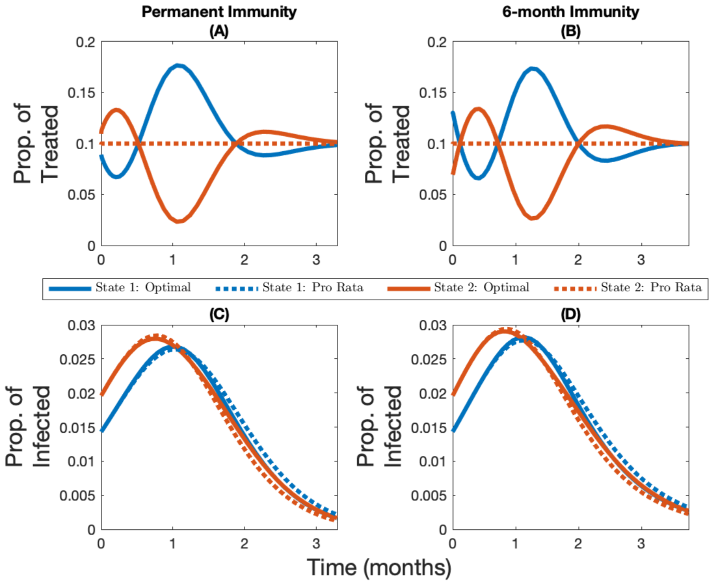

Figure S6: **Compliance to travel restrictions with permanent and temporary immunity.** Change over time in the optimal and pro rata allocations (panels A and B) and the corresponding infection levels (panels C and D) for State 1 (in blue, the initially lowest-burdened state) and State 2 (in red, the initially highest-burdened state) depending on whether immunity is permanent (panels A and C) or lasts six months (panels B and D) for the case where the vaccine capacity constraint is 10% and there is compliance to travel restrictions.

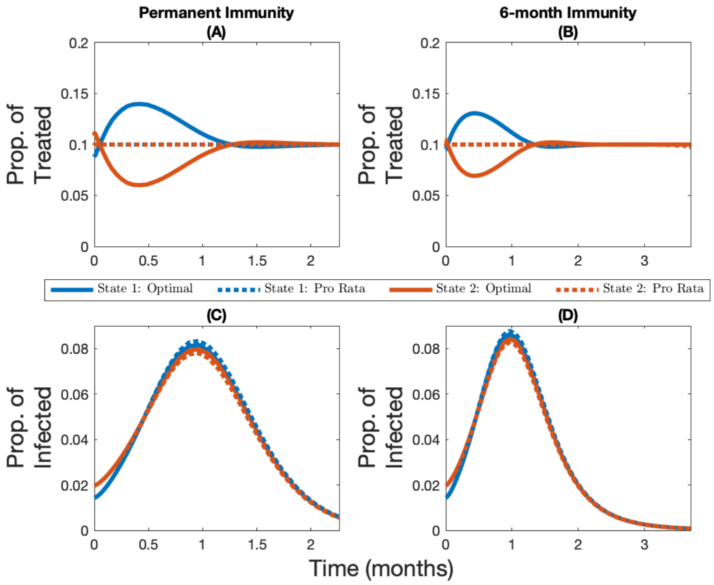

Figure S7: **Noncompliance to travel restrictions with permanent and temporary immunity.** Change over time in the optimal and pro rata allocations (panels A and B) and the corresponding infection levels (panels C and D) for State 1 (in blue, the initially lowest-burdened state) and State 2 (in red, the initially highest-burdened state) depending on whether immunity is permanent (panels A and C) or lasts six months (panels B and D) for the case where the vaccine capacity constraint is 10% and there is no compliance to travel restrictions.

C.1.5 Cumulative Infection Levels

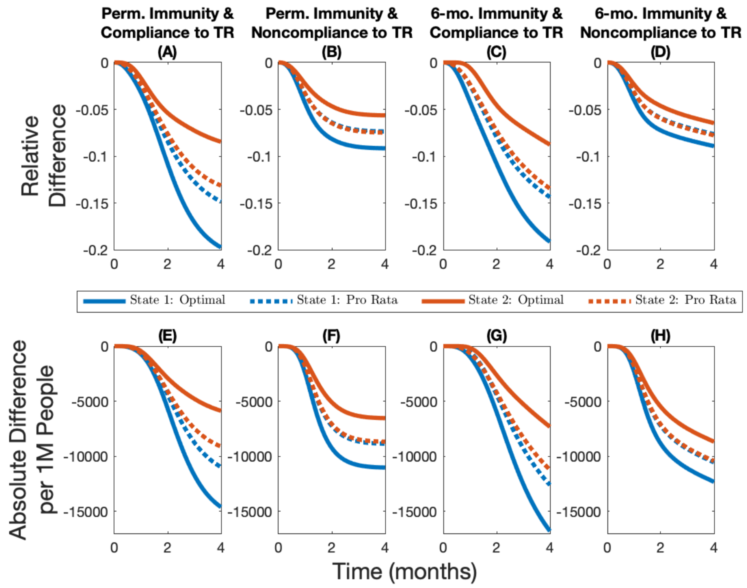

Figure S8: **Epidemiological outcomes under different scenarios with a low vaccine supply.** Cumulative relative difference (panels A, B, C, and D) and cumulative absolute difference per 1M people (panels E, F, G, and H) between the number of infections in different allocations rules and the no-vaccine case for different immunity–travel restrictions scenarios and for when vaccine capacity is 5%.

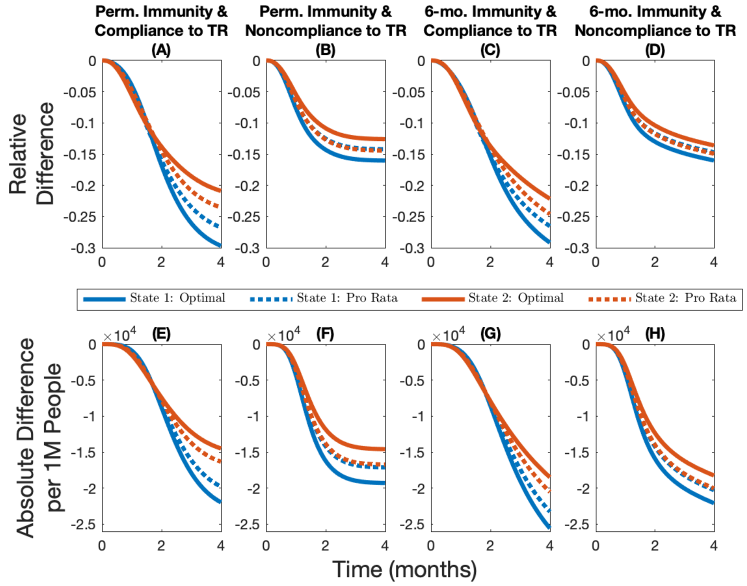

Figure S9: **Epidemiological outcomes under different scenarios with vaccines.** Cumulative relative difference (panels A, B, C, and D) and cumulative absolute difference per 1M people (panels E, F, G, and H) between the number of infections in different allocations rules and the no-vaccine case for different immunity–travel restrictions (TR) scenarios and for when vaccine capacity is 10%.

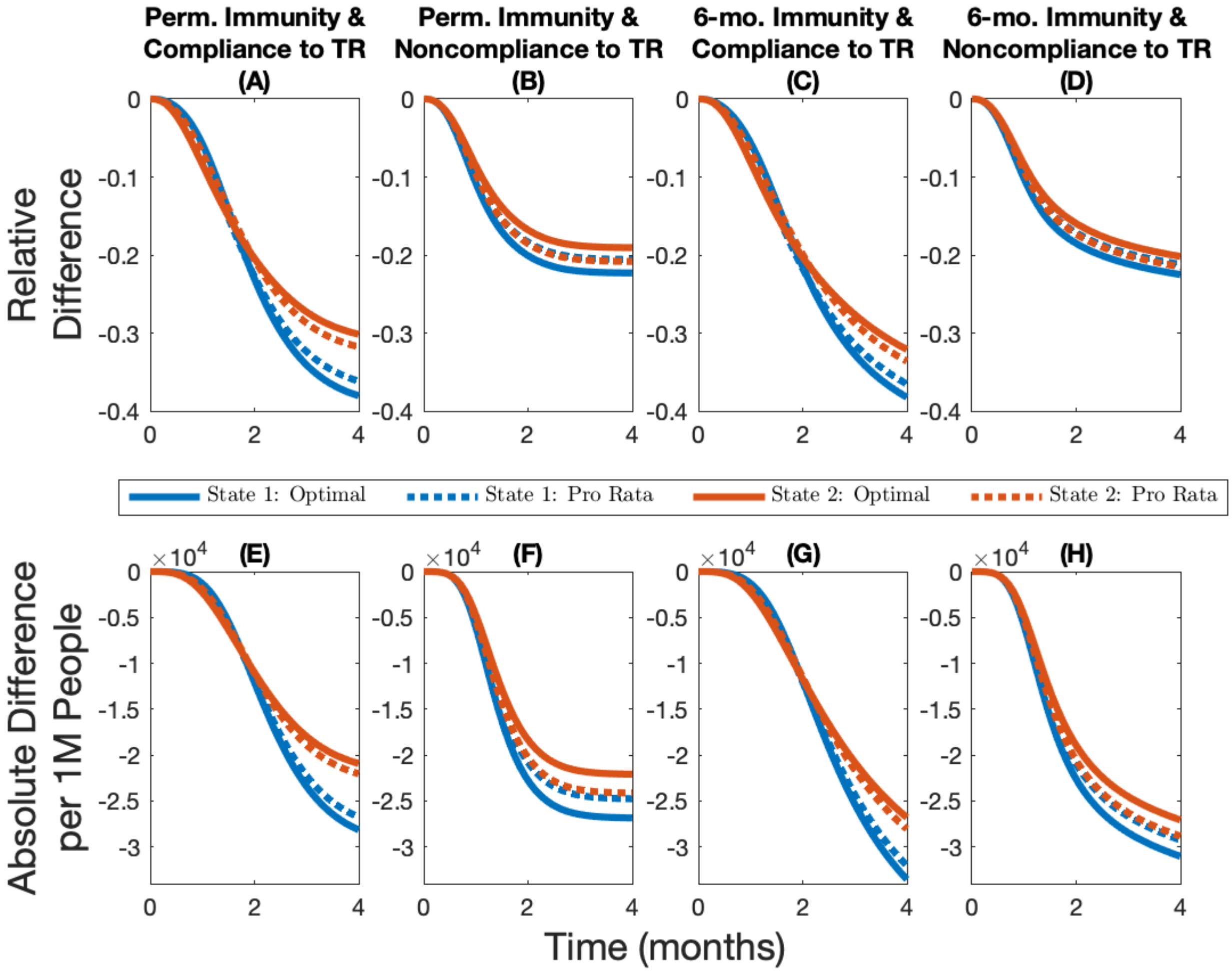

Figure S10: **Epidemiological outcomes under different scenarios with a high vaccine supply.** Cumulative relative difference (panels A, B, C, and D) and cumulative absolute difference per 1M people (panels E, F, G, and H) between the number of infections in different allocations rules and the no-vaccine case for different immunity–travel restrictions scenarios and for when vaccine capacity is 15%.

C.2 Heterogeneous Demographic Characteristics

C.2.1 Heterogeneous Case-Fatality Ratio

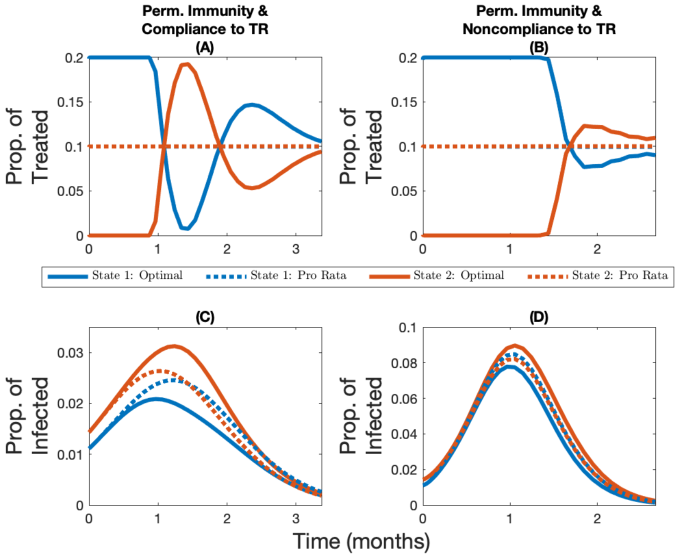

Figure S11: **Vaccine allocation with and without compliance to travel restrictions.** Change over time in the optimal and pro rata allocations (panels A and B) and the corresponding infection levels (panels C and D) for State 1 (in blue, the initially lowest-burdened state) and State 2 (in red, the initially highest-burdened state) depending on whether there is compliance to travel restrictions (panels A and C) or not (panels B and D) for the case where the vaccine capacity constraint is 10%, immunity is permanent, and where the heterogeneity in the system comes from a varying case-fatality ratio (State 1 has a case-fatality ratio 1 percentage point higher than State 2).

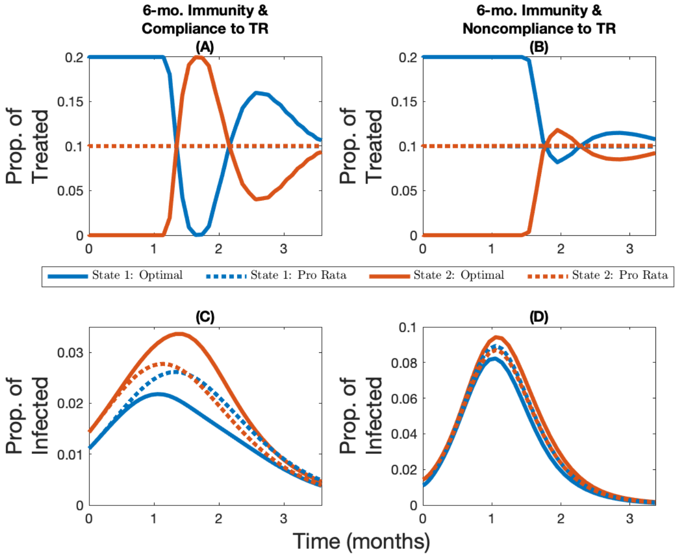

Figure S12: **Vaccine allocation with and without compliance to travel restrictions.** Change over time in the optimal and pro rata allocations (panels A and B) and the corresponding infection levels (panels C and D) for State 1 (in blue, the initially lowest-burdened state) and State 2 (in red, the initially highest-burdened state) depending on whether there is compliance to travel restrictions (panels A and C) or not (panels B and D) for the case where the vaccine capacity constraint is 10%, immunity lasts six months, and where the heterogeneity in the system comes from a varying case-fatality ratio (State 1 has a case-fatality ratio 1 percentage point higher than State 2).

C.2.2 Heterogeneous Contact Rate

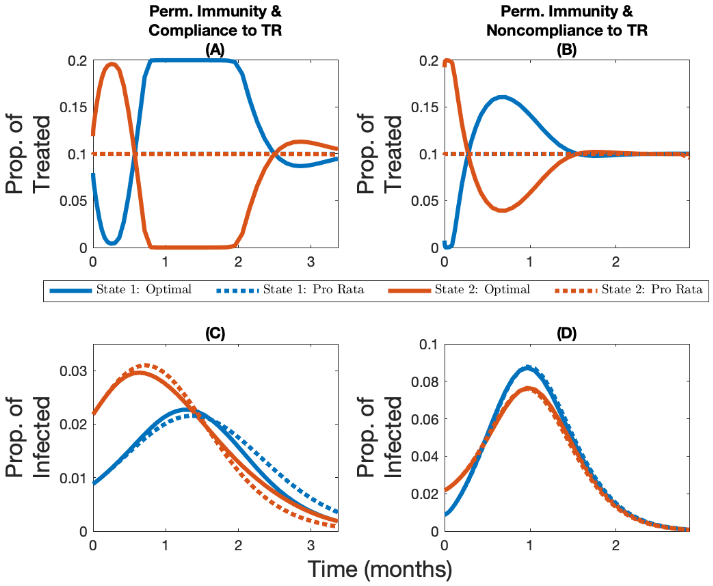

Figure S13: **Vaccine allocation with and without compliance to travel restrictions.** Change over time in the optimal and pro rata allocations (panels A and B) and the corresponding infection levels (panels C and D) for State 1 (in blue, the initially lowest-burdened state) and State 2 (in red, the initially highest-burdened state) depending on whether there is compliance to travel restrictions (panels A and C) or not (panels B and D) for the case where the vaccine capacity constraint is 10%, immunity is permanent, and where the heterogeneity in the system comes from a varying contact rate (State 2 has a higher contact rate than State 1).

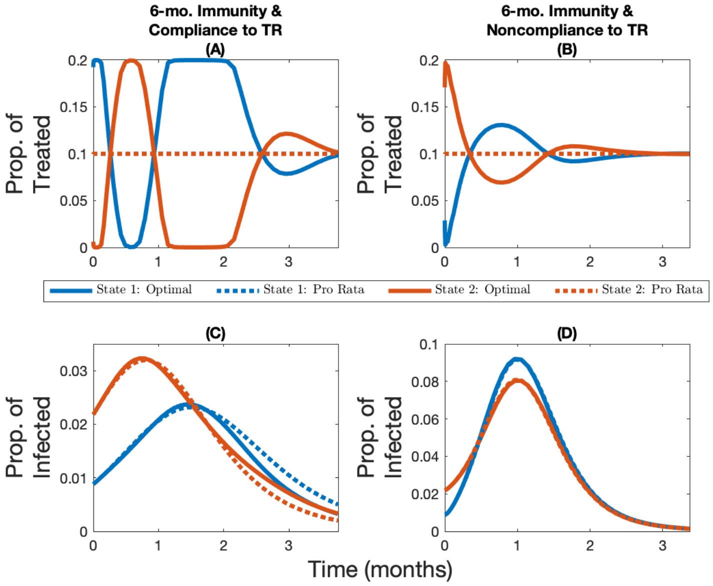

Figure S14: **Vaccine allocation with and without compliance to travel restrictions.** Change over time in the optimal and pro rata allocations (panels A and B) and the corresponding infection levels (panels C and D) for State 1 (in blue, the initially lowest-burdened state) and State 2 (in red, the initially highest-burdened state) depending on whether there is compliance to travel restrictions (panels A and C) or not (panels B and D) for the case where the vaccine capacity constraint is 10%, immunity lasts six months, and where the heterogeneity in the system comes from a varying contact rate (State 2 has a higher contact rate than State 1).

C.3 Robustness of Spatial allocations

C.3.1 Homogeneous Demographic Characteristics

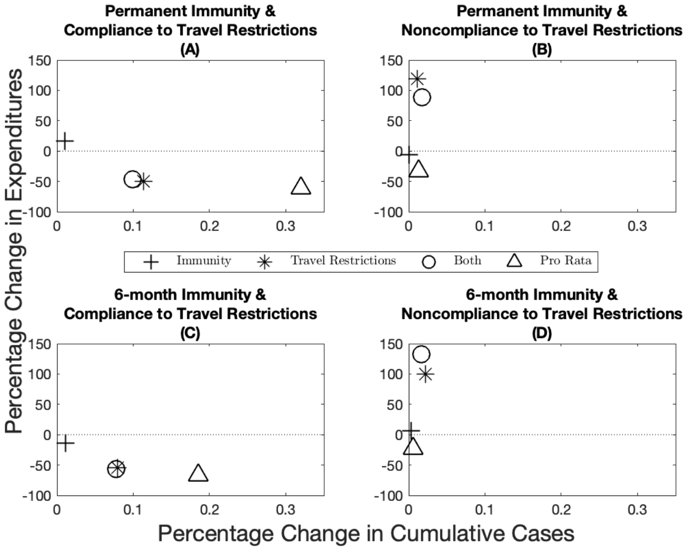

Figure S15: **Robustness of epidemiological and economic outcomes under different scenarios.** Percentage change in expenditures (y-axis) and percentage change in cumulative cases (x-axis) from the optimal allocation for different immunity–travel restrictions scenarios and for when vaccine capacity is 5%.

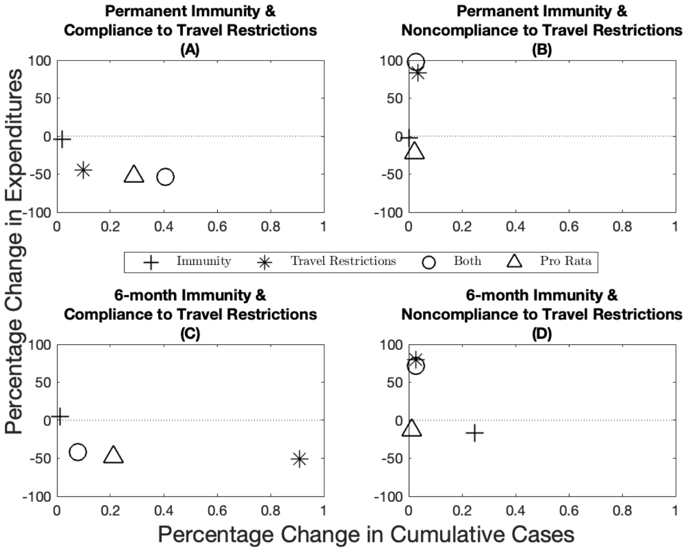

Figure S16: **Robustness of epidemiological and economic outcomes under different scenarios.** Percentage change in expenditures (y-axis) and percentage change in cumulative cases (x-axis) from the optimal allocation for different immunity–travel restrictions scenarios and for when vaccine capacity is 15%.

C.3.2 Heterogeneous Case-Fatality Ratio

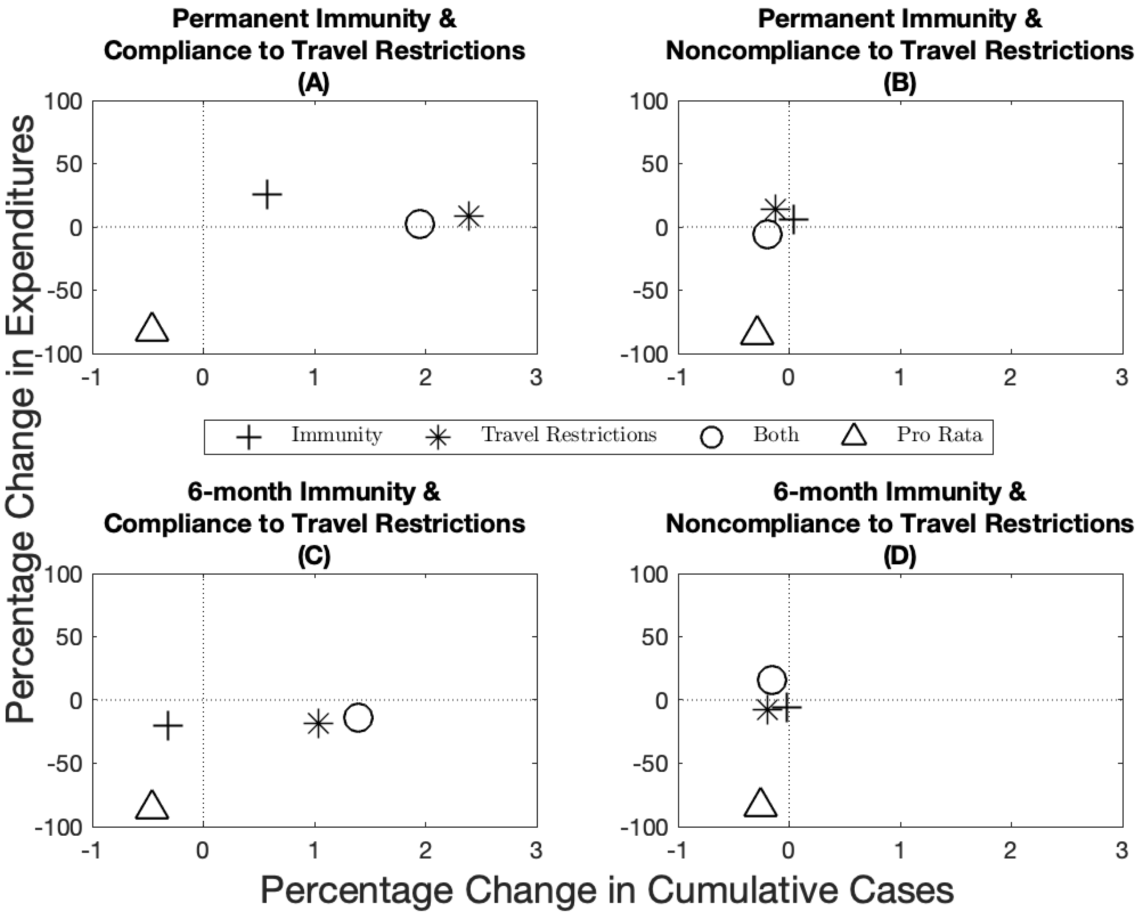

Figure S17: **Robustness of epidemiological and economic outcomes under different scenarios when the source of heterogeneity is the case-fatality ratio.** Percentage change in expenditures (y-axis) and percentage change in cumulative cases (x-axis) from the optimal allocation for different immunity–travel restrictions scenarios and for when vaccine capacity is 10%.

C.4 Sensitivity Analyses

C.4.1 Workability Cost

As mentioned above, imposing the pro rata rule *ex ante* implicitly means that the central planning agency is essentially assuming that the cost of deviating from it is infinite. In practice, the workability cost is hard to quantify, because it depends on logistical, political, and cultural factors. It does however seem reasonable to assume, as we did in the paper, that the cost is finite. We investigate the sensitivity of our results by solving for the optimal vaccine allocation over time with levels lower and higher than the base case parameter in the paper. We summarize these results by plotting the variance of the optimal deviation from the pro rata rule in each time period (in blue; Figure S18 panels A, B, C, and D), and the difference in cumulative cases between the optimal and pro rata allocations (in red; Figure S18 panels A, B, C, and D) as we vary the scale of the workability cost. The variance is calculated as $\text{Var}\left(\frac{\text{Optimal Vaccine}-\text{Pro Rata Vaccine}}{\text{Pro Rata Vaccine}}\right)$. Note that the variance of the optimal deviation from the pro rata rule is identical in absolute and relative terms across jurisdictions. Mathematically, as the workability cost approaches zero, the optimal control problem becomes linear in the controls, which implies that there is no adjustment cost associated with changing the allocation. Often times this can lead to extreme solutions (allocation goes to one state for a time period and then the other state, and so on).

Given the behavior and nature of the problem, therefore, we expect that at lower values of the workability cost parameter we will find higher variance of the deviation. This in turn results in a higher performance of the optimal allocation relative to the pro rata rule, in terms of reduction in cumulative cases. When we increase the workability cost parameter, the cost parameter will eventually be on the same magnitude as the VSL (Figure S18 black line represents the VSL). When we reach levels this high, the optimal allocation converges towards the pro rata rule and any differences in cumulative cases disappear.

We also show how amount of funds allocated to the workability cost over time compare to expenditures on the total vaccine cost (Figure S18 panels E, F, G, and H). If this ratio exceeds one, the planner is spending on aggregate more to deviate from the pro rata rule than on treatments. These panels show that at low levels of the workability cost parameter, the total workability costs are small relative to the total vaccine costs. As the workability cost parameter increases, however, the total workability costs become more and more important relative to the total vaccine cost. Eventually, these costs begin to dominate the planners objective and the deviation between the pro rata rule and optimal allocations goes to zero.

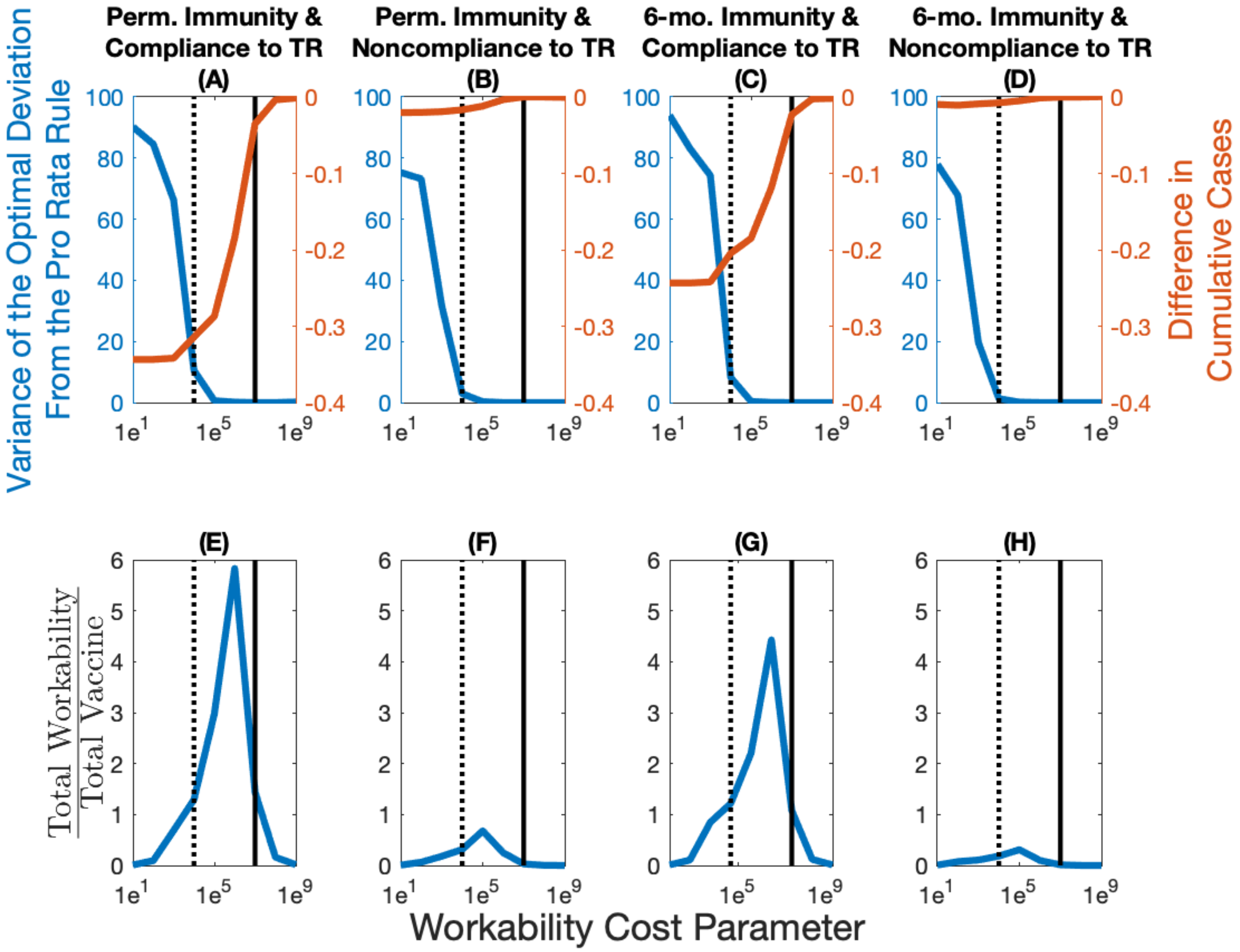

Figure S18: **Sensitivity of optimal allocations and epidemiological and economic outcomes when varying the workability cost parameter.** The variance of the optimal deviation in percentage (in blue; panels A, B, C, and D) represents an aggregate measure of the optimal deviation from the pro rata allocation. The difference in cumulative cases between the optimal and pro rata allocations (in red; panels A, B, C, and D) represents in percentage terms how well the optimal allocation outperforms the pro rata allocation. The total workability cost over the total vaccine cost (panels E, F, G, and H) represents how many times more the total workability costs are relative to the total vaccine costs. The dotted vertical line represents the base case value of the workability cost parameter ($1e^4$), while the full vertical line represents the value of statistical life ($1e^7$).

C.4.2 Vaccine Effectiveness

The base case parameter for vaccine effectiveness we utilized in the paper is based on estimates of the influenza vaccine (43); see Appendix A for more details. Recent evidence from the COVID-19 vaccines suggest that effectiveness could be considerably higher. As a result, we investigate how a more effective vaccine would affect the nature of our results. We find that the more effective a vaccine is, the more a central planner would want to deviate from the pro rata rule (in blue; Figure S19 panels A, B, C, and D). As a result of this greater deviation, we see a larger difference in terms of the reduction in cumulative cases (in red; Figure S19 panels A, B, C, and D). Because a higher effectiveness results in a greater deviation, then, everything else equal, the total workability costs are increased relative to the total vaccine costs (Figure S19 panels E, F, G, and H). The differences are more stark in a world where there is compliance to travel restrictions, as noncompliance blurs the spatial heterogeneity across the jurisdictions leading in general to allocations similar to the pro rata rule.

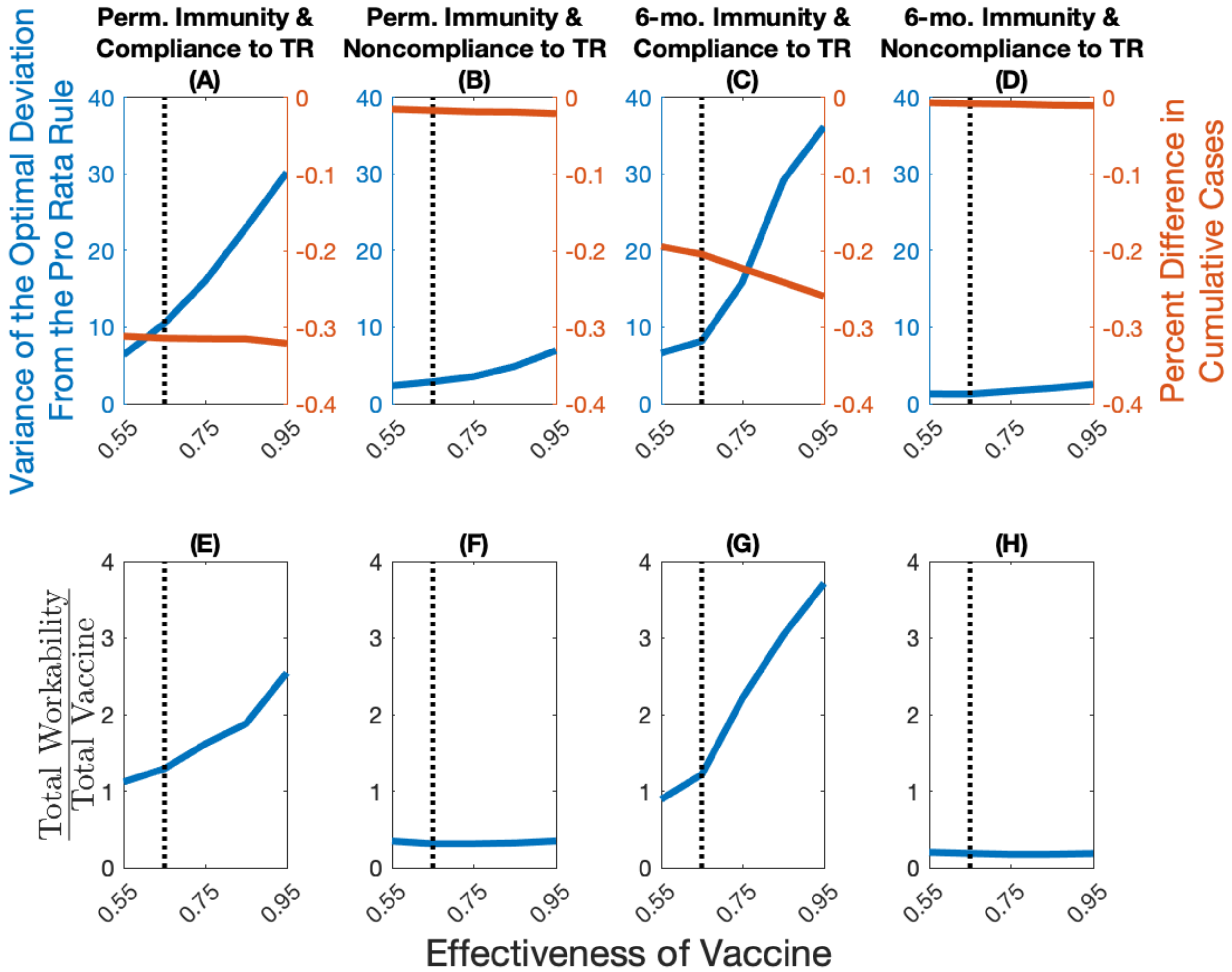

Figure S19: **Sensitivity of optimal allocations, and of epidemiological and economic outcomes when varying the effectiveness of the vaccine.** The variance of the optimal deviation in percentage (in blue; panels A, B, C, and D) represents an aggregate measure of the optimal deviation from the pro rata rule. The difference in cumulative cases between the optimal and pro rata allocations (in red; panels A, B, C, and D) represents in percentage terms how well the optimal allocation outperforms the pro rata rule. The total workability cost over the total vaccine cost (panels E, F, G, and H) represents how many times more the total workability costs are relative to the total vaccine costs. The dotted vertical line in the plots represents the base case value of the vaccine effectiveness (0.65).
